# Functional interdependence of the actin regulators CAP1 and cofilin1 in control of dendritic spine morphology

**DOI:** 10.1007/s00018-022-04593-8

**Published:** 2022-10-20

**Authors:** Anika Heinze, Cara Schuldt, Sharof Khudayberdiev, Bas van Bommel, Daniela Hacker, Toni G. Schulz, Ramona Stringhi, Elena Marcello, Marina Mikhaylova, Marco B. Rust

**Affiliations:** 1 Molecular Neurobiology Group, Institute of Physiological Chemistry, Philipps-University of Marburg, 35032 Marburg, Germany; 2 Center for Mind, Brain and Behavior (CMBB), University of Marburg and Justus-Liebig-University Giessen, 35032 Marburg, Germany; 3 AG Optobiology, Institute of Biology, Humboldt-University, 10115 Berlin, Germany; 4 Guest Group ‘Neuronal Protein Transport’, Institute for Molecular Neurogenetics, Center for Molecular Neurobiology (ZMNH), University Medical Center Hamburg-Eppendorf (UKE), 20251 Hamburg, Germany; 5 Department of Pharmacological and Biomolecular Sciences, Università degli Studi di Milano, 20133 Milan, Italy; 6 DFG Research Training Group ‘Membrane Plasticity in Tissue Development and Remodeling’, GRK 2213, Philipps-University of Marburg, 35032 Marburg, Germany; 7 Institute of Chemistry and Biochemistry, Department of Biology, Chemistry and Pharmacy, Freie Universität Berlin, 14195 Berlin, Germany

**Keywords:** Actin dynamics, Actin turnover, Synaptic actin, Synaptic plasticity, Postsynaptic density

## Abstract

The vast majority of excitatory synapses are formed on small dendritic protrusions termed dendritic spines. Dendritic spines vary in size and density that are crucial determinants of excitatory synaptic transmission. Aberrations in spine morphogenesis can compromise brain function and have been associated with neuropsychiatric disorders. Actin filaments (F-actin) are the major structural component of dendritic spines, and therefore, actin-binding proteins (ABP) that control F-actin dis-/assembly moved into the focus as critical regulators of brain function. Studies of the past decade identified the ABP cofilin1 as a key regulator of spine morphology, synaptic transmission, and behavior, and they emphasized the necessity for a tight control of cofilin1 to ensure proper brain function. Here, we report spine enrichment of cyclase-associated protein 1 (CAP1), a conserved multidomain protein with largely unknown physiological functions. Super-resolution microscopy and live cell imaging of CAP1-deficient hippocampal neurons revealed impaired synaptic F-actin organization and dynamics associated with alterations in spine morphology. Mechanistically, we found that CAP1 cooperates with cofilin1 in spines and that its helical folded domain is relevant for this interaction. Moreover, our data proved functional interdependence of CAP1 and cofilin1 in control of spine morphology. In summary, we identified CAP1 as a novel regulator of the postsynaptic actin cytoskeleton that is essential for synaptic cofilin1 activity.

**Supplementary Information:**

The online version contains supplementary material available at 10.1007/s00018-022-04593-8.

## Introduction

Most excitatory synapses in the vertebrate brain are formed on small dendritic protrusions termed dendritic spines [7]. Dendritic spines contain a postsynaptic density (PSD), which opposes the presynaptic active zone and consists of scaffolding proteins anchoring neurotransmitter receptors, ion channels, adhesion, and signaling molecules that collectively mediate postsynaptic responses to neurotransmitter release [66]. The PSD scaffold is directly linked to the underlying actin cytoskeleton. Actin filaments (F-actin) are the major cytoskeletal component in spines, and actin-binding proteins (ABP) that regulate assembly or disassembly of F-actin control spine morphology and density [7]. Consequently, dysfunction of ABPs affects synaptic transmission and has been associated with mental retardation or neuropsychiatric diseases, such as autism spectrum disorder (ASD), attention-deficit/hyperactivity disorder (ADHD), or schizophrenia [8, 21, 68]. Hence, deciphering postsynaptic actin regulatory mechanisms is mandatory to understand the processes that control brain function and that contribute to pathologies of human neuropsychiatric diseases.

Studies of the past decade unraveled important synaptic functions for actin depolymerizing proteins of the ADF/cofilin family [6, 10, 11, 15, 17, 22, 52, 55, 57, 69, 74, 78]. Specifically, these studies identified cofilin1 as a key regulator of postsynaptic F-actin dynamics. Further, they implicated cofilin1 in the pathology of neuropsychiatric diseases and proved modulation of cofilin1 activity as a promising therapeutic avenue. Together, these studies highlighted the necessity for a tight regulation of synaptic cofilin1 activity to ensure brain function [3, 55, 56]. De-/phosphorylation of a conserved serine residue at position 3 (Ser3) is as an important mechanism to control cofilin1 activity, and several pathways that impinge on either LIM kinases or slingshot, which phosphorylate or dephosphorylate Ser3, respectively, have been identified to date [55]. However, apart from these pathways, only very little is known about synaptic cofilin1 regulation.

By using recombinant proteins, recent in vitro work established the multidomain protein cyclase-associated protein1 (CAP1) as a regulator of F-actin dynamics [25, 29, 30, 65]. These studies implicated the conserved helical folded domain (HFD) in actin dissociation from F-actin and β-sheets within the CAP and RP2 (CARP) domain in nucleotide exchange on globular actin monomers (G-actin). However, the physiological functions of CAP1 remained largely unknown [58, 59]. Here, we report enrichment of CAP1 in the head of dendritic spines. Depletion of CAP1 in primary hippocampal neurons from gene-targeted mice revealed a role for CAP1 in regulating the postsynaptic actin cytoskeleton as well as spine morphology and density. We show that this depended on its helical folded domain (HFD), which is relevant for the interaction with cofilin1. Rescue experiments in double knockout neurons lacking CAP1 and cofilin1 revealed their cooperation in the regulation of spine shape and they proved functional interdependence of both ABP in the postsynaptic compartment. Together, we found CAP1 to be important for cofilin1 function in spines, and we thereby report a novel mechanism that controls synaptic cofilin1 activity.

## Results

### CAP1 is expressed in the postnatal brain and enriched in dendritic spines

**Fig. 1:**
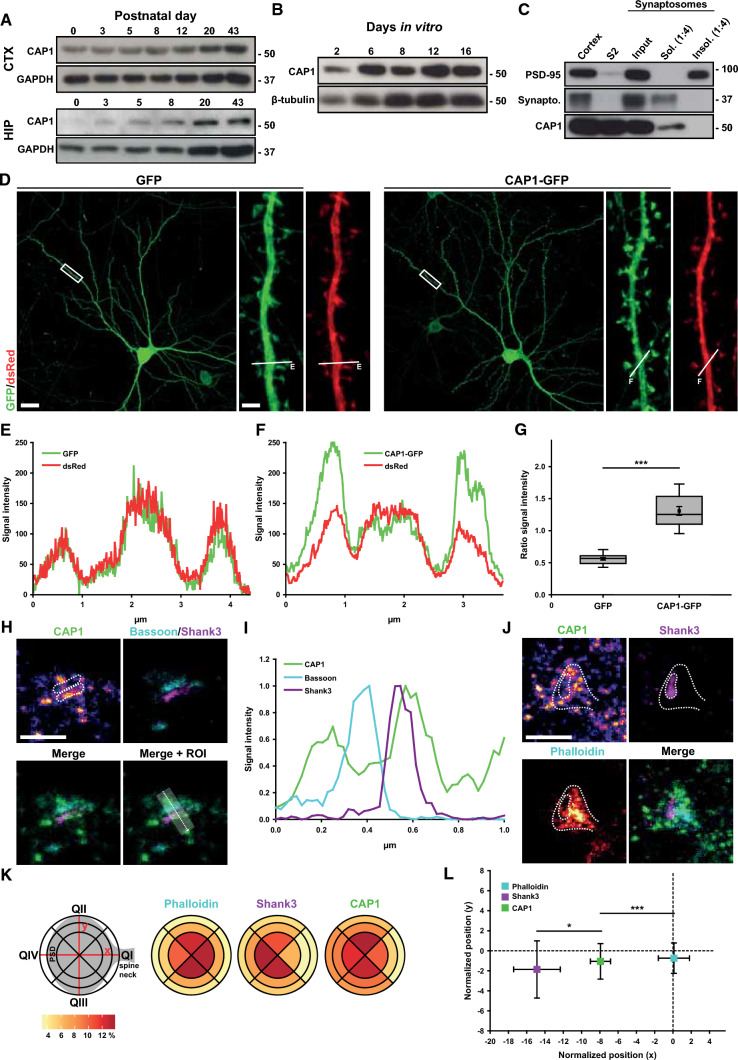
HYPERLINK "sps:id::fig1||locator::gr1||MediaObject::0" Enrichment of CAP1 in spine heads. **A** Immunoblots showing CAP1 expression throughout postnatal cerebral cortex (CTX) and hippocampal (HIP) development. GAPDH was used as loading control. **B** Immunoblots showing CAP1 expression in isolated cerebral cortex neurons. β-tubulin was used as loading control. **C** Immunoblots showing the presence of CAP1 in synaptosomes and in the soluble (Sol.), but not in the insoluble protein fraction (Insol.). PSD-95 and synaptophysin (Synapto.) proved separation of protein fractions. **D** DIV16 hippocampal neurons expressing the volume marker dsRed (red) together with either GFP or CAP1-GFP (green). Boxes indicate areas shown at higher magnification. **E** Fluorescence intensity profiles for GFP and dsRed along line E in **D**. **F** Fluorescence intensity profiles for GFP and dsRed along line F in **D**. **G** Box plots (incl. mean values (MV) ± standard error of the means (SEM)) of GFP ratio in spine heads vs. dendritic shafts in neurons expressing either GFP or CAP1-GFP. **H** STED images of an excitatory synapse from a neuron stained with antibodies against CAP1 (‘fire’ (single channel), green (merge)), the presynaptic marker Bassoon (cyan), and the PSD marker Shank3 (magenta). Areas framed by dotted lines in upper left image indicate distribution of Bassoon and Shank3. **I** Integrated fluorescence intensity profiles for CAP1, Bassoon, and Shank3 along transparent box shown in **H**, direction is indicated by dashed arrow. **J** STED images of a dendritic spine from a neuron stained with antibodies against CAP1 (‘fire’ (single channel), green (merge)) and Shank3 (magenta) as well as the F-actin marker phalloidin (‘red hot’ (single channel), cyan (merge)). Areas framed by dotted lines in upper left image indicate distribution of phalloidin and Shank3. **K** Graphs showing relative distribution of CAP1, Shank3, and phalloidin in spine heads. Scheme on the left shows the mask that was used for this analysis. **L** Graph showing center of mass for CAP1, Shank3, and phalloidin in spines. Coordinate system’s origin indicates spine center (see scheme in **K**). Scale bars (µm): 1 (**H**, **J**), 2 (**D**, high magnification), 20 (**D**, low magnification). *P < 0.05, ***P < 0.001

While previous studies reported the presence of CAP1 during embryonic brain development [4, 61], its expression in the postnatal brain and its subcellular localization in differentiated neurons are unknown. Immunoblots revealed the presence of CAP1 throughout postnatal development in mouse cerebral cortex and hippocampus (Fig. 1A). Similarly, CAP1 was expressed in mouse primary neurons kept in culture for 2–16 days (Fig. 1B). Further, we found CAP1 present in the soluble, but not in the insoluble, fraction of synapse-enriched lysates termed synaptosomes (Fig. 1C). Together, CAP1 is expressed throughout postnatal brain development, and our data suggest synaptic localization.

To further study the subcellular localization of CAP1, we exploited mouse hippocampal neurons isolated at embryonic day (E) 18.5. We transfected neurons after 6 days in vitro (DIV) with constructs expressing green fluorescent protein (GFP)-tagged CAP1 (CAP1-GFP) and the volume marker *Discosoma* red fluorescent protein (dsRed), and we determined CAP1-GFP localization at DIV16 (Fig. 1D). In control neurons, we expressed GFP that showed the expected equal distribution within the dendritic compartment, similar to the volume marker dsRed (Fig. 1D, E). CAP1-GFP was present in virtually all dendritic spines, independent of their size or morphology. Compared to GFP-expressing neurons, GFP intensity was increased in spines of neurons expressing CAP1-GFP (Fig. 1D). This was also evident from the higher GFP intensity in spines when compared to dsRed (GFP/dsRed ratio) for both mushroom-like and thin spines (Figs. 1F, S1A). These data suggested enrichment of CAP1 in dendritic spines, which was confirmed by a 2.3-fold increase in the ratio of GFP intensity in mushroom-like spines vs. dendritic shafts in CAP1-GFP neurons (Fig. 1G; GFP: 0.56 ± 0.02, CAP1-GFP: 1.31 ± 0.07, *n* = 150 spines from 20 neurons from three hippocampal cultures, *P* < 0.001). Supportively, GFP intensity profile in CAP1-GFP-expressing neurons overlapped with that of the F-actin marker mCherry-LifeAct, which as expected was enriched in dendritic spines (Fig. S1B, C). Antibody staining confirmed CAP1 presence in dendritic spines and proved that CAP1-GFP faithfully reflects localization of endogenous protein (Fig. S1D). Hippocampal neurons from brain-specific CAP1-KO mice were exploited for control experiments [61], which proved specificity of the antibody (Fig. S1E).

To determine the sub-synaptic localization of endogenous CAP1, we performed stimulated emission depletion (STED) nanoscopy, which confirmed CAP1 localization in spines and unveiled partial colocalization with the PSD marker Shank3 (Figs. 1H, I; S2). Additionally, it revealed CAP1 localization in presynaptic boutons identified by Bassoon immunoreactivity. To better characterize CAP1 distribution in spines, we performed STED nanoscopy on neurons stained with Shank3 and the F-actin marker phalloidin (Figs. 1J, S3), which were used to outline the PSD and spines, respectively. We quantified relative fluorescence of CAP1, Shank3 and phalloidin by using a mask that subdivides spine heads into quadrants (Q), each of which were composed of three equally sized segments (Fig. 1K), similar to a previous study [41]. As expected, Shank3 was enriched in QIV, opposite to the spine neck, and phalloidin intensity was equally high in QI-III, but low in QIV. While CAP1 was present in all four quadrants, it was rather weak in the region close to the spine neck (QI) and showed highest intensity in QII and III. These data were in line with partial colocalization of CAP1 and Shank3, but suggested higher CAP1 abundance in spine head regions outside the PSD. We also determined the centers of mass within spines for all three fluorescent signals. As shown in the scheme (Fig. 1K), the coordinate system’s origin represents the center of the circular region of interest. As expected, this analysis revealed no differences between Shank3, phalloidin and CAP1 along the y-axis (Fig. 1L; Shank3: −1.86 ± 2.41, phalloidin: −0.74 ± 1.29, CAP1: −1.05 ± 1.50, *n* = 20/3; Shank3 vs CAP1: *P* = 0.812, phalloidin vs CAP1: *P* = 0.893, Shank3 vs phalloidin: *P* = 0.730). CAP1’s center of mass along the *x*-axis, which approximately reflected the PSD-spine neck axis, laid between Shank3 and phalloidin and differed from both (CAP1: −7.93 ± 0.93; phalloidin: 0.11 ± 1.44, *P* < 0.001; Shank3: −14.88 ± 2.15, *P* < 0.05), thereby supporting our assumption that CAP1 is enriched in spine head regions beneath the PSD. Together, our data revealed localization of endogenous CAP1 in both pre- and postsynaptic compartments of hippocampal excitatory synapses. In the postsynaptic compartment, CAP1 was enriched in spine head regions underneath the PSD.

### CAP1 is relevant for dendritic spine density and morphology

To investigate the neuronal function of CAP1, we genetically removed CAP1 from hippocampal neurons isolated from conditional CAP1 (CAP1^flx/flx^) mice [61]. We therefore transfected CAP1^flx/flx^ neurons at DIV6 with either catalytically active mCherry-Cre (Cre) or a catalytically inactive mutant Cre variant (Cre-mut). Immunocytochemistry proved absence of CAP1 from Cre-transfected CAP1^flx/flx^ neurons (Fig. S4), which we refer to as CAP1-KO neurons throughout the manuscript. Instead, CAP1 was present upon Cre-mut transfection, and these neurons served as control (CTR). First, we performed a thorough morphometric analysis of GFP-transfected neurons at DIV16 (Fig. S5A). In CAP1-KO neurons, the number of primary neurites, the number of branching points or the branching point number normalized to dendritic length was unchanged (Fig. S5B-D, Tab. S1), suggesting overall preserved morphology. To better judge dendritic tree complexity, we performed Sholl analysis, which revealed a slightly lower number of intersections between concentric circles and dendrites at intermediate distances from soma in CAP1-KO neurons (Fig. S5E, F). While the radius with highest count of intersections was closer to the soma in CAP1-KO neurons, the maximal number of intersections and the ramification index (maximal intersections/primary dendrites) was not different between CTR and CAP1-KO neurons (Fig. S5G–I, Tab. S1). Together, morphology was largely preserved in CAP1-KO neurons, and they displayed only slight changes in dendritic tree complexity.

**Fig. 2:**
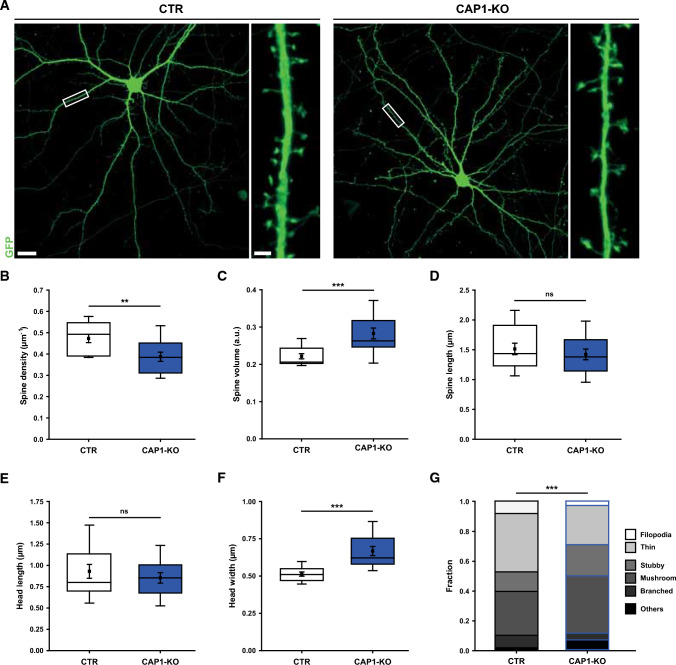
Reduced spine density and increased spine size in CAP1-KO neurons. **A** Micrographs of CTR and CAP1-KO neurons expressing GFP (green). Boxes indicate areas shown at higher magnification. Box plots (incl. MV ± SEM) of **B** total spine density, **C** spine volume, **D** spine length, **E** spine head length, and **F** spine head widths in CTR and CAP1-KO neurons. **G** Stacked column graph showing fractions of spine types in CTR and CAP1-KO neurons. Scale bar (µm): 2 (high magnification) 20 (low magnification). ns: *P* ≥ 0.05, **P < 0.01, ***P < 0.001

CAP1 enrichment in dendritic spines motivated us to next determine spine number and morphology. Compared to CTR, spine density was reduced by 17%, while spine volume was increased by 27% (Fig. 2A–C, Tab. S2). Consistent with enlarged spine volume, spine head width was increased by 31%, while total spine length or spine head length were unchanged (Fig. 2D–F, Tab. S3). Further, we determined the fractions of spine types in CAP1-KO neurons (Fig. S6A), similar to previous studies [20]. In CAP1-KO neurons, the fractions of filopodia-like and thin spines were reduced, while the fractions of stubby and mushroom-like spines were increased (Fig. 2G; Tab. S4). Hence, the spine type distribution was shifted towards larger spines (stubby, mushroom-like) in CAP1-KO neurons. Together, CAP1 inactivation increased spine size and reduced spine density. Since F-actin constitutes the major structural component in spines and determines their morphology, we hypothesize a role for CAP1 in regulating the postsynaptic actin cytoskeleton.

### CAP1 controls the postsynaptic actin cytoskeleton

**Fig. 3:**
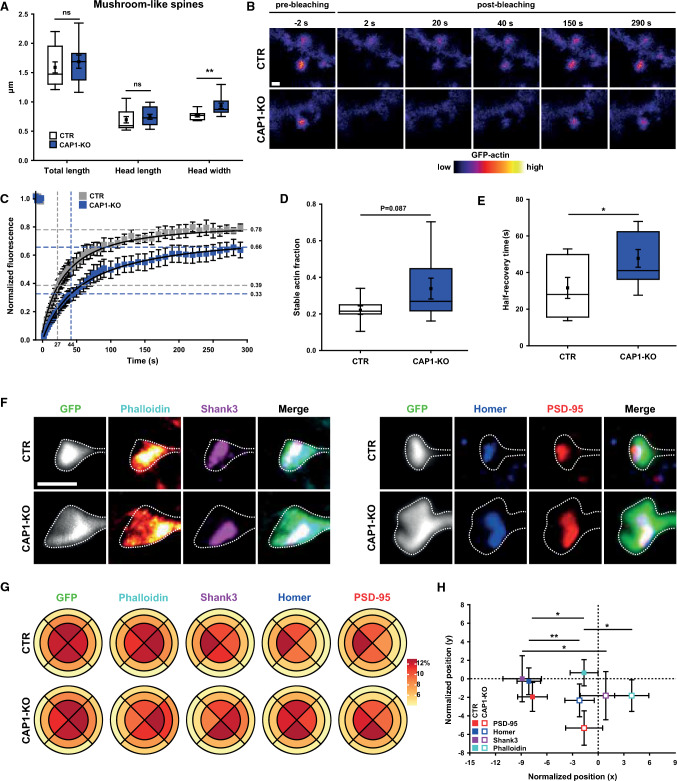
CAP1 controls actin turnover and F-actin distribution in spines. **A** Box plots (incl. MV ± SEM) showing morphometric analysis of mushroom-like spines in CTR and CAP1-KO neurons. **B** Image sequence of mushroom-like spines from CTR and CAP1-KO neurons expressing GFP-actin during fluorescence recovery after photobleaching (FRAP). **C** GFP-actin recovery curve in mushroom-like spines from CTR and CAP1-KO neurons. Normalized fluorescence recovery after 300 s (plateau, *y*-axis) as well as half-recovery time (*x*-axis) based on 50% of the plateau (*y*-axis) are indicated by gray lines for CTR and by blue lines for CAP1-KO neurons. Box plots (incl. MV ± SEM) showing **D** stable actin fraction and **E** half-recovery time of GFP-actin in CTR and CAP1-KO spines. **F** STED images showing mushroom-like spines of GFP-expressing CTR and CAP1-KO neurons. Neurons were stained with either an antibody against Shank3 (magenta) or phalloidin (‘red hot’ (single channel), cyan (merge)) or with antibodies against Homer (blue) and PSD-95 (red). GFP is shown in grayscale (single channel) or green (merge) as indicated. **G** Relative distribution of fluorescence intensities of phalloidin, Shank3, PSD-95, and Homer in CTR and CAP1-KO spines. GFP was used to determine spine morphology. Relative fluorescence intensities were plotted for each segment as shown in Fig. 1K. **H** Graph showing centers of mass for phalloidin, Shank3, PSD-95, and Homer in CTR and CAP1-KO spines. Centers of mass were normalized to GFP. Scale bars (µm): 1 (**B**, **F**). ns: *P* ≥ 0.05, *P < 0.05, **P < 0.01

To test this hypothesis, we investigated both postsynaptic actin turnover and F-actin organization in CAP1-KO neurons. For better comparability, we restricted our analysis to mushroom-like spines, which displayed a 22% increase in head width in CAP1-KO neurons (Fig. 3A, Tab. S3), while the morphologies of filopodia-like, thin, or stubby spines were unchanged (Fig. S6B–D, Tab. S3). To study whether CAP1 was relevant for actin turnover, we expressed GFP-actin in neurons and performed fluorescence recovery after photobleaching (FRAP) experiments in individual spines (Fig. 3B, Movies S1-2). During 300 s of recording, GFP-actin levels in CTR spines reached a plateau at roughly 0.8 of basal levels (Fig. 3C, D), and the stable actin fraction that did not recover within 300 s amounted to 0.22 ± 0.02 (*n* = 32/8/3). While this fraction was 54% higher in CAP1-KO spines (0.34 ± 0.06, *n* = 33/9/3), this increase did not reach statistical significance. However, compared to CTR, fluorescence recovery was slower in CAP1-KO spines, as evident from a 51% increase in half-recovery time of the dynamic actin fraction (Fig. 3E; (s) CTR: 31.60 ± 5.76, CAP1-KO: 47.75 ± 4.83, *P* < 0.05). Hence, fluorescence recovery of GFP-actin was slowed down in CAP1-KO spines, demonstrating a role for CAP1 in postsynaptic actin turnover.

Next, we tested whether impaired actin turnover was associated with an altered F-actin organization. We therefore performed STED nanoscopy on fixed GFP-expressing neurons stained with phalloidin (Figs. 3F, S7). GFP was used to outline spines and, thus, to determine relative distribution of fluorescence intensities of phalloidin as shown in the scheme in Fig. 1K. In CTR spines, we found the expected homogeneous distribution of phalloidin in QI-III and lower levels in QIV (Fig. 3G). Compared to CTR, phalloidin distribution was changed in CAP1-KO spines, in which it was enriched in QI, at the spine head base. Hence, relative F-actin distribution was altered in CAP1-KO spines. Because F-actin in spines is relevant for anchoring scaffolding proteins of the PSD, we next determined whether altered F-actin distribution in CAP1-KO spines was associated with an altered distribution of the key PSD proteins Shank3, PSD-95, and Homer (Figs. 3F, S7–8). As expected, all three scaffolding proteins were enriched in QIV in CTR spines (Fig. 3G). Such enrichment was not present in CAP1-KO spines. We also determined the centers of mass for phalloidin and the PSD proteins (Fig. 3H). To exclude potential discrepancies due to differences in spine size between CTR and CAP1-KO spines, we normalized the centers of mass to that of GFP, which corresponded to the coordinate system’s origin (scheme in Fig. 1K). Consistent with a homogeneous distribution of phalloidin in QI-III from CTR spines, its center of mass was close to the origin with a *x*-value of −1.65 ± 1.63 (*n* = 26). The centers of mass for PSD-95, Shank3, and Homer in CTR spines were found further on the left, as expected from their enrichment in QIV (*x*-values: PSD-95: −7.68 ± 1.74, *n* = 30; Shank3: −8.88 ± 2.25, *n* = 26; Homer: −8.11 ± 1.38, *n* = 57). Compared to CTR, the centers of mass were different for phalloidin and all three PSD proteins in CAP1-KO spines, thereby confirming an altered distribution of F-actin and PSD proteins (*x*-values: phalloidin: 3.91 ± 2.00, *P* < 0.05, *n* = 29; PSD-95: −1.64 ± 2.16, *P* < 0.05, *n* = 24; Shank3: 0.89 ± 2.99, *P* < 0.05, *n* = 29; Homer: −2.18 ± 1.72, *P* < 0.001, *n* = 47). Hence, our data revealed an altered F-actin distribution within CAP1-KO spines, suggesting a role for CAP1 in organizing the postsynaptic actin cytoskeleton. Interestingly, F-actin disorganization was associated with an altered distribution of key PSD proteins. Together, we identified CAP1 as a novel regulator of the postsynaptic actin cytoskeleton relevant for both actin dynamics and F-actin organization.

### HFD and CARP domain are relevant for CAP1 function in spines

**Fig. 4:**
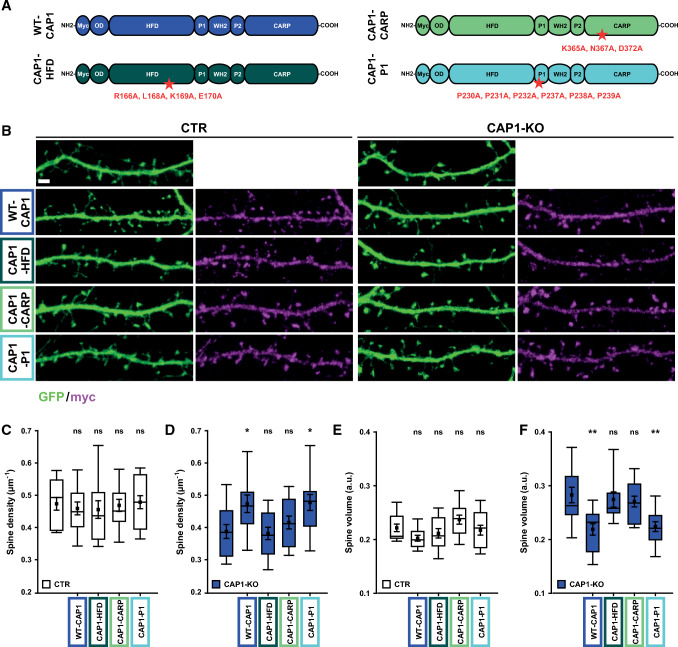
Helical folded domain and CARP domain are relevant for CAP1 function in spines. **A** Schemes showing domain structure of myc-tagged CAP1 as well as the mutations introduced into CAP1-HFD, CAP1-CARP, and CAP1-P1. OD: oligomerization domain, HFD: helical folded domain, P1: proline-rich domain 1, WH2: Wiskott-Aldrich syndrome homology domain 2, P2: proline-rich domain 2, CARP: CAP and retinitis pigmentosa protein 2 domain. **B** Micrographs showing GFP and antibody staining against myc in CTR and CAP1-KO neurons expressing myc-tagged WT-CAP1 or mutant variants. Box plots (incl. MV ± SEM) showing spine density **C** in CTR or **D** in CAP1-KO neurons and spine volume **E** in CTR or **F** in CAP1-KO neurons upon expression of WT-CAP1 or mutant CAP1 variants. Asterisks and ns indicate significance of changes when compared to CTR neurons (**C**, **E**) or CAP1-KO neurons (**D**, **F**). Scale bar (µm): 2. ns: *P* ≥ 0.05, *P < 0.05, **P < 0.01

Having established CAP1 as a novel postsynaptic actin regulator, we next set out to determine the CAP1-dependent mechanism. Site-directed mutagenesis implicated its HFD and CARP domain in regulating actin dynamics [29, 30]. To test the relevance of these domains in spines, we expressed myc-tagged CAP1 variants (WT-CAP1, CAP1-HFD, and CAP1-CARP) together with the volume marker GFP and either Cre-mut or Cre in CAP1^flx/flx^ neurons (Fig. 4A). These experiments also included a CAP1 variant with mutations in a proline-rich domain (CAP1-P1) that reportedly disrupted interaction with the ABP profilin [5, 37]. Myc antibody staining confirmed successful triple transfection of CAP1^flx/flx^ neurons (Fig. S9), and it revealed localization of all mutant variants and of myc-WT-CAP1 in spines from CTR and CAP1-KO neurons (Fig. 4B). Neither WT-CAP1 nor mutant CAP1 variants altered spine density or volume in CTR neurons. Conversely, expression of WT-CAP1 increased spine density by 20% in CAP1-KO neurons, and it reduced spine volume by 21% (Fig. 4C–F, Tab. S2). Both parameters were not different from CTR neurons (*P* = 0.993 and *P* = 0.825, respectively). Hence, WT-CAP1 rescued spine density and morphology in CAP1-KO neurons to CTR values. Similarly, CAP1-P1 normalized both parameters in CAP1-KO neurons. Instead, neither CAP1-HFD nor CAP1-CARP changed spine density or volume in CAP1-KO neurons. We therefore concluded that HFD and CARP domain, but not the proline-rich domain P1, were relevant for CAP1 function in spines.

### *CAP1 and cofilin1 interact *in vitro* and in cells*

**Fig. 5:**
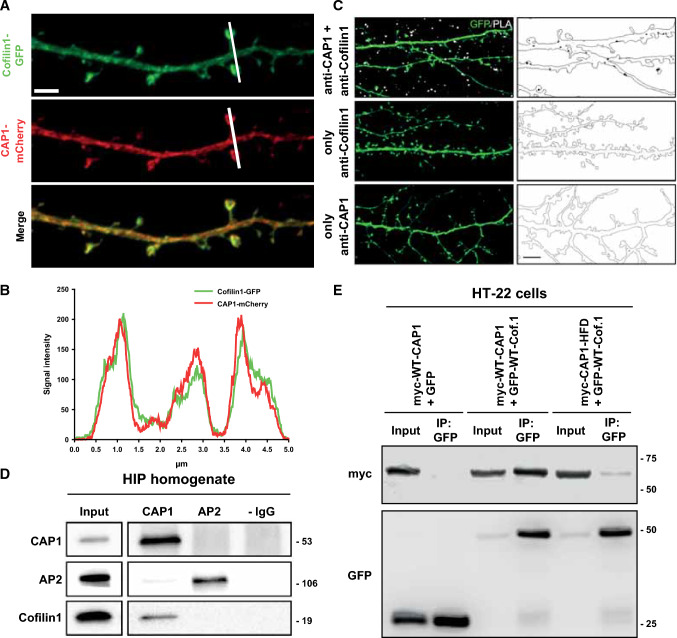
Physical interaction of CAP1 and cofilin1 in hippocampal neurons. **A** Dendritic shaft of a hippocampal neuron expressing cofilin1-GFP (green) and CAP1-mCherry (red). **B** Fluorescence intensity profiles for cofilin1-GFP and CAP1-mCherry along white line in **A**. Left-to-right direction in graph corresponds to top-to-bottom direction in micrograph. **C** Micrographs of hippocampal neurons used for proximity ligation assay (PLA) to show colocalization of endogenous CAP1 and cofilin1. Neurons expressed GFP (green) that served as a volume marker to outline dendritic compartment (line in black–white images). PLA signal (white dots in left images, black dots in right images) was present in neurons stained with antibodies against both ABP, but not in neurons stained with an antibody against CAP1 or cofilin1 alone, thereby confirming specificity of PLA signal. **D** Immunoblot analysis of proteins precipitated with either a mouse monoclonal CAP1 antibody or a mouse monoclonal antibody recognizing the β2-adaptin subunit of AP2 from hippocampal (HIP) homogenates. Cofilin1 coprecipitated with CAP1, but not with AP2—IgG: no IgG. **E** Immunoblots with antibodies against myc and GFP in lysates from HT-22 cells expressing either (i) myc-WT-CAP1 and GFP, (ii) myc-WT-CAP1 and GFP-WT-cofilin1 or (iii) myc-CAP1-HFD and GFP-WT-cofilin1. In the presence of GFP-WT-cofilin1, an antibody against GFP precipitated myc-WT-CAP1, but not myc-CAP1-HFD. Scale bars (µm): 2 (**A**), 5 (**C**)

Since previous studies suggested a role for CAP1’s HFD and CARP domain in cofilin1-dependent actin dynamics [29, 30, 57], we next tested whether or not CAP1 was relevant for the function of cofilin1, which emerged as a key postsynaptic actin regulator [6, 17, 19, 22, 53, 55, 57]. Immunoblots revealed increased expression of both ABP during postnatal brain development (Fig. S10A-C), and fluorescence intensity profiles in neurons expressing CAP1-mCherry and cofilin1-GFP suggested colocalization in dendritic spines (Fig. 5A, B). Indeed, colocalization of both ABPs in the dendritic compartment was confirmed in proximity ligation assays (PLA) by exploiting antibodies against endogenous CAP1 and endogenous cofilin1 (Fig. 5C). Further, cofilin1 was precipitated from mouse hippocampal lysates with a mouse monoclonal antibody against CAP1, but not with a mouse monoclonal antibody against the β2-adaptin subunit of adaptor protein 2 (AP2) that was used as a negative control (Fig. 5D). These findings suggested physical interaction of both ABPs, which we confirmed in a mouse hippocampal neuronal cell line. In these experiments, we expressed myc-WT-CAP1 together with either GFP or GFP-WT-cofilin1 in HT-22 cells. Myc-WT-CAP1 was precipitated with an antibody against GFP in lysates from GFP-WT-cofilin1-expressing HT-22 cells, but not from GFP-expressing HT-22 cells (Fig. 5E). Moreover, hardly any myc-CAP1-HFD was precipitated in lysates from HT-22 cells co-transfected with GFP-WT-cofilin1, demonstrating the relevance of HFD for the interaction with cofilin1. Together, colocalization of CAP1 and cofilin1 as well as their interaction in hippocampal neurons let us hypothesize that both ABPs cooperate in spines.

### CAP1 and cofilin1 cannot compensate the other’s inactivation in spines

**Fig. 6:**
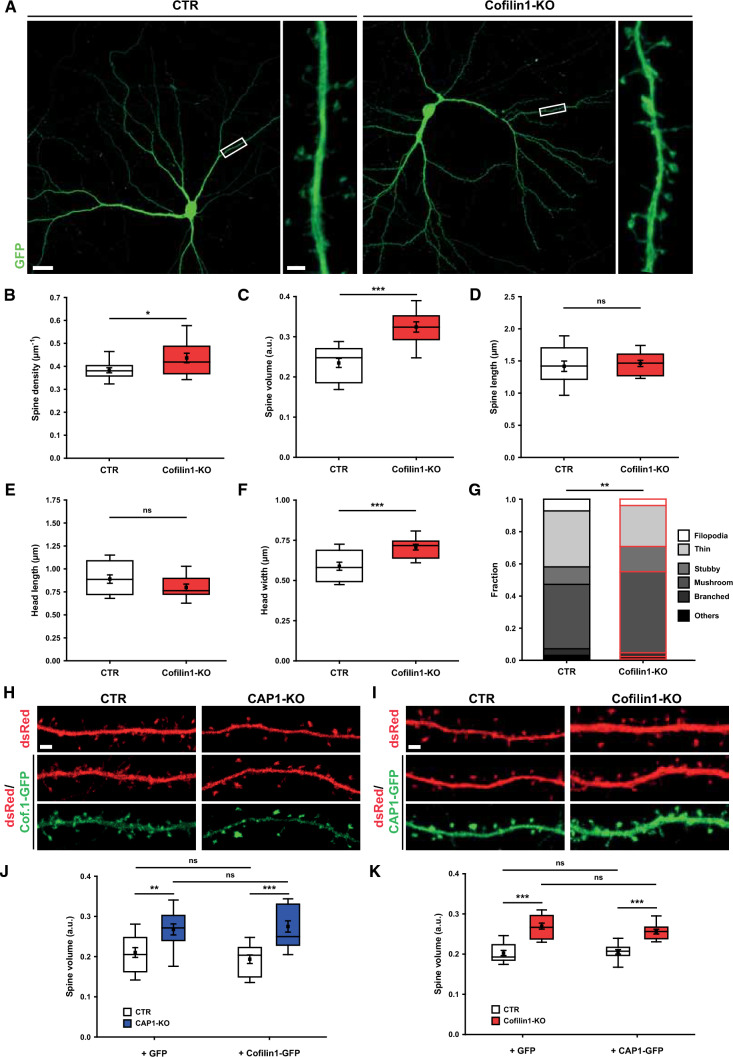
Cofilin1-KO neurons display increases in spine size and fraction of large spines. **A** Micrographs of GFP-expressing CTR and cofilin1-KO neurons. Boxes indicate areas shown at higher magnification. Box plots (incl. MV ± SEM) showing **B** spine density, **C** spine volume, **D** spine length, **E** spine head length, and **F** spine head widths in CTR and cofilin1-KO neurons. **G** Stacked column graph showing fractions of spine types in CTR and cofilin1-KO neurons. **H** Micrographs of dendritic shafts from CTR and CAP1-KO neurons transfected with dsRed (red) together with either GFP (not shown) or cofilin1-GFP (green). **I** Micrographs of dendritic shafts from CTR and cofilin1-KO neurons transfected with dsRed (red) together with either GFP (not shown) or CAP1-GFP (green). **J** Box plots (incl. MV ± SEM) showing spine volume in CTR and CAP1-KO neurons either expressing GFP or cofilin1-GFP. **K** Box plots (incl. MV ± SEM) showing spine volume in CTR and cofilin1-KO neurons either expressing GFP or CAP1-GFP. Scale bar (µm): 2 (**A**, high magnification, **H**, **I**), 20 (**A**, low magnification). ns: *P* ≥ 0.05, *P < 0.05, **P < 0.01, ***P < 0.001

To test this hypothesis, we compared under identical experimental conditions spine defects in CAP1-KO neurons to those in neurons lacking cofilin1. We generated cofilin1-KO neurons by Cre expression in hippocampal neurons from E18.5 Cfl1^flx/flx^ mice [2], Cre-mut-expressing Cfl1^flx/flx^ neurons served as CTR. Cofilin1-KO neurons displayed increased spine density and volume (Fig. 6A–C, Tab. S2), similar to spine changes reported for hippocampal neurons in fixed sections from cofilin1-KO mice [57]. Further analysis of cofilin1-KO neurons showed a 20% increase in spine head width, while total spine length or head length were unchanged, similar to CAP1-KO neurons (Fig. 6D–F, Tab. S3). While morphometric analysis of individual spine types did not reveal differences between CTR and cofilin1-KO neurons (Fig. S11A–D, Tab. S3), the spine type distribution in cofilin1-KO neurons was shifted towards larger spines (Fig. 6G, Tab. S4). Together, cofilin1-KO neurons displayed an increase in spine volume and an increased fraction of large spines, very similar to CAP1-KO neurons.

Next, we tested whether CAP1 and cofilin1 were able to compensate the other’s inactivation in spines. To do so, we overexpressed WT-cofilin1 in CAP1-KO neurons and vice versa WT-CAP1 in cofilin1-KO neurons (Fig. 6H, I). Overexpression of WT-cofilin1 neither restored spine volume nor density in CAP1-KO neurons (Figs. 6J, S11E, Table S5). Likewise, WT-CAP1 overexpression did not alter spine density or volume in cofilin1-KO neurons (Figs. 6K, S11F, Tab. S5). Together, CAP1 and cofilin1 failed in compensating the other’s absence in spines, thereby supporting our hypothesis that both ABPs cooperate in spines.

### CAP1 and cofilin1 are functionally interdependent in spines

**Fig. 7:**
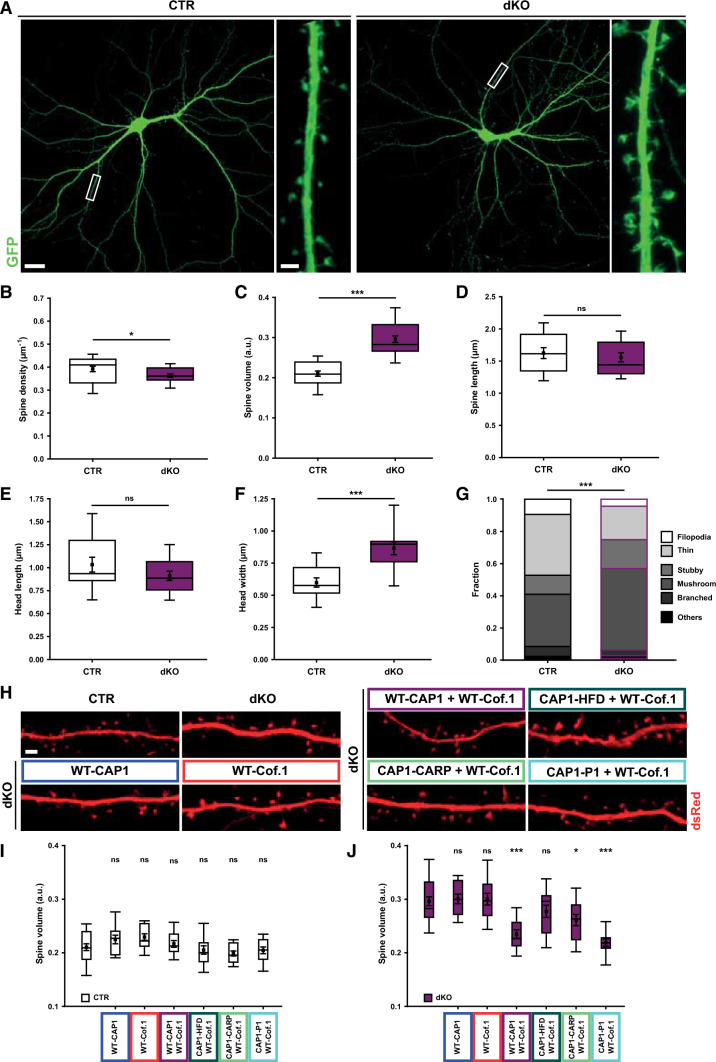
Cooperation of CAP1 and cofilin1 in spines. **A** Micrographs of GFP-expressing CTR and double-KO (dKO) neurons. Boxes indicate areas shown at higher magnification. Box plots (incl. MV ± SEM) showing **B** spine density, **C** spine volume, **D** spine length, **E** spine head length, and **F** spine head width in CTR and dKO neurons. **G** Stacked column graph showing fraction of spine types in CTR and dKO neurons. **H** Micrographs showing dsRed in CTR neurons as well as in dKO neurons upon transfection of CAP1 and/or cofilin1 constructs as indicated. Box plots (incl. MV ± SEM) showing spine volume **I** in CTR and **J** in dKO neurons upon expression of WT-cofilin1 and/or CAP1 constructs as indicated. Asterisks and ns indicate significance of changes when compared to CTR neurons (**I**) or dKO neurons (**J**). Scale bars (µm): 2 (**A**, high magnification, **H**), 20 (**A**, low magnification). ns: *P* ≥ 0.05, *P < 0.05, ***P < 0.001

To test this hypothesis, we analyzed double KO (dKO) neurons lacking both ABPs, which we achieved by Cre expression in CAP1^flx/flx^/Cfl1^flx/flx^ neurons. Cre-mut-expressing CAP1^flx/flx^/Cfl1^flx/flx^ neurons served as CTR (Fig. 7A). Different from CAP1-KO or cofilin1-KO neurons, spine density was only very slightly reduced in dKO neurons (Fig. 7B, Tab. S6). However, spine volume was increased by roughly 40% (Fig. 7C, Tab. S6). Notably, spine volume in dKO neurons did not differ from CAP1-KO or cofilin1-KO neurons (Tab. S6). Hence, compound inactivation of both ABPs did not cause an additive effect in spine volume.

A detailed spine morphometric analysis in dKO neurons revealed a 45% increase in spine head width, while total spine length or head length were unchanged (Fig. 7D–F, Tab. S3). While morphology of filopodia-like and stubby spines was normal in dKO (Fig. S12A–D, Tab. S3), thin spine length was reduced by 14%, and head width of mushroom-like spines was increased by 22%. Spine categorization according to their morphology revealed a shift towards larger spines in dKO neurons (Fig. 7G, Tab. S4). Together, spine size and the fraction of large spines were increased in dKO neurons, very similar to those changes we found in CAP1-KO and cofilin1-KO neurons.

**Fig. 8:**
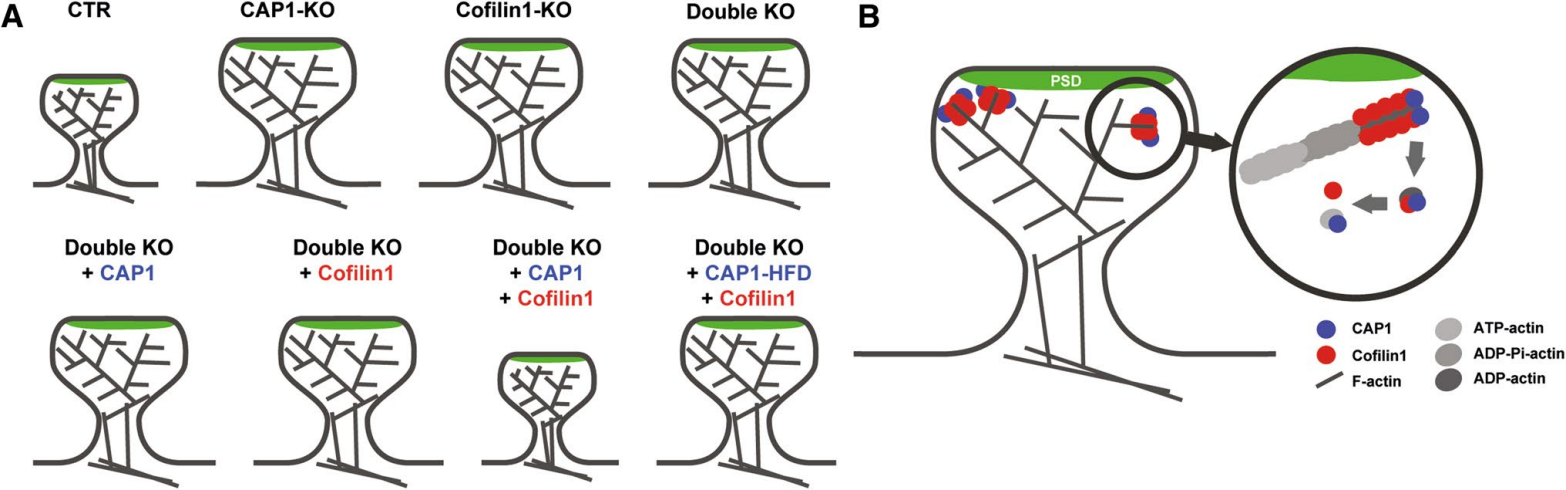
Functional interdependence of CAP1 and cofilin1 in regulating dendritic spine size. **A** Scheme showing spine enlargement in neurons lacking CAP1, cofilin1, or both ABP as well as normalization of spine size in double KO neurons upon expression of WT-CAP1 and cofilin1, but not upon expression of CAP1-HFD and cofilin1. **B** The present study provides evidence for an interaction of the actin regulators CAP1 and cofilin1 in control of dendritic spine morphology. Our data and previous in vitro studies that unraveled a cooperation of CAP1 with cofilin1 in actin regulation [29, 30] let us hypothesize a cooperation of CAP1 and cofilin1 in postsynaptic actin regulation as well as functional interdependence of both ABP

To finally test whether CAP1 and cofilin1 require each other to elicit their effects in spines, we expressed either WT-CAP1, WT-cofilin1 or both ABP in dKO neurons and determined spine volume as a readout. Neither WT-CAP1 nor WT-cofilin1 reduced spine volume in dKO neurons (Fig. 7H–J, Tab. S6). Instead, spine volume in dKO neurons was rescued upon co-expression of WT-CAP1 and WT-cofilin1. We also expressed WT-cofilin1 together with aforementioned CAP1 mutants in dKO neurons and found that spine volume was rescued upon expression with CAP1-P1. While expression of WT-cofilin1 and CAP1-CARP slightly reduced spine volume in dKO neurons, expression of WT-cofilin1 together with CAP1-HFD failed to normalize spine volume. In summary, these data demonstrated that CAP1 and cofilin1 cooperated in the regulation of spine morphology. Further, they revealed functional interdependence of CAP1 and cofilin1, which depends on CAP1’s HFD (Fig. 8).

## Discussion

The ABP CAP1 is a multidomain protein with largely unknown physiological functions. Here we report CAP1 expression throughout postnatal brain development and enrichment in dendritic spine heads. By STED microscopy and live cell imaging in hippocampal neurons from gene-targeted mice, we identified CAP1 as a novel postsynaptic actin regulator relevant for spine density and morphology. Mechanistically, we found the conserved HFD to be essential for CAP1’s function in spines and for its interaction with the key synaptic actin regulator cofilin1. Rescue experiments in dKO lacking CAP1 and cofilin1 revealed that both ABP not only cooperated in regulating spine morphology but also demonstrated their mutual functional dependence in the postsynaptic compartment.

The yeast CAP ortholog has been recognized as an ABP three decades ago (for review: [46, 58, 59], but significant progress in its molecular functions has been achieved only recently, predominantly by exploiting recombinant proteins or mutant yeast strains [24, 25, 29, 30, 65]. In a current model of actin regulation, CAP interacts with cofilin-decorated F-actin via its HFD and thereby accelerates dissociation of the terminal actin subunit [29, 30]. Subsequently, G-actin-cofilin complexes are passed on to CAP’s CARP domain that, together with its WH2 domain, releases cofilin from this complex and promotes nucleotide (ATP for ADP) exchange on G-actin, which is required for actin polymerization. While these studies provided exciting novel insights into CAP’s molecular activities, the cellular and physiological functions of mammalian CAP largely remained unknown, also because appropriate animal models were lacking. This held true specifically for CAP1, while earlier mouse studies implicated CAP2 in heart physiology and skeletal muscle development [9, 12, 28, 47]. Recently, a TALEN-engineered systemic CAP1-KO mouse model has been generated, but these mice died during embryonic development and analysis of heterozygous mutants solely revealed a role of CAP1 in lipoprotein metabolism [23]. Moreover, we recently reported impaired neuron connectivity in brain-specific CAP1-KO mice, which was likely caused by compromised growth cone function and delayed neuron differentiation [61, 63]. Instead, the function of CAP1 in differentiated neurons or synapses has not been studied to date.

In the present study, we demonstrated important functions for CAP1 in postsynaptic actin regulation, in very good agreement with the in vitro studies outlined above. Specifically, FRAP revealed a role for CAP1 in postsynaptic actin turnover and STED nanoscopy in postsynaptic F-actin organization. Interestingly, actin defects were associated with an altered sub-spinous distribution of the PSD proteins PSD-95, Shank3 and Homer, thereby suggesting a role for CAP1 in shaping the postsynaptic machinery, which will be investigated in future studies. While we here implicated CAP1 in synaptic actin regulation, we earlier showed its relevance for actin dynamics in growth cones [61, 63]. We therefore conclude a general requirement of CAP1 in neuronal actin regulation both during differentiation and in differentiated neurons, similar to cofilin1 [2, 14, 45, 55, 57, 62]. By PLA we found colocalization (within 40 nm) of CAP1 and cofilin1 in the dendritic compartment, and our co-immunoprecipitation experiments validated their physical interaction in the hippocampus, which required CAP1’s HFD. By rescue experiments in dKO neurons lacking CAP1 and cofilin1, we demonstrated a cooperation of both ABP in regulating spine morphology. Moreover, we showed mutual functional dependence of CAP1 and cofilin1 in spines, similar to their functional interdependence in growth cones [61]. Hence, an intimate interaction of CAP1 and cofilin1 is likely of general relevance for neuronal actin regulation.

While this study reports an important synaptic function for CAP1, studies of the past two decades recognized cofilin1 as an important regulator of synapse physiology, synaptic plasticity, brain function and behavior [6, 13, 17, 22, 56, 57, 60, 77]. In line with these publications, we report increased spine density and volume in cofilin1-KO neurons. Collectively, these studies let cofilin1 emerge as a key regulator of spine morphology and as a major final point of signaling output for actin regulation in spines [68]. This emphasized the necessity for regulatory mechanisms that tightly control cofilin1 activity to ensure proper synapse physiology and brain function. In fact, dysregulation of cofilin1 activity has been linked to synaptic and behavioral deficits associated with ASD or ADHD [10, 78]. To date, a plethora of signaling molecules ranging from the Rho GTPases Rac1, Cdc42, and RhoA and their effectors PAK1, ROCK, and LIMK1 to the phosphatase calcineurin and its effectors PI3K and slingshot have been implicated in synaptic cofilin1 phosphorylation that controls actin binding (for review: [3, 56, 68]. Additionally, synaptic cofilin1 activity is regulated by molecules that control its recruitment into spines and by translation within the dendritic compartment [11, 49, 51]. By demonstrating mutual functional dependence of cofilin1 and CAP1 in spines, we report a conceptually novel mechanism of synaptic cofilin1 regulation. Further, our finding that CAP1 was essential for cofilin1 activity in spines opened up a new avenue for the modulation of cofilin1 activity and, hence, actin dynamics in spines. Notably, CAP1 comprises several conserved domains allowing interaction with molecules others than actin and cofilin1. To date, a number of interaction partners have been found for CAP1 or its homologs (for review: [26, 46, 58, 59], including established regulators of spine morphology, such as the ABP profilin [1, 32, 39, 40, 70], the proteinase MMP-9 [71, 73], the tyrosine kinases Abl1 and Abl2 [33, 36, 44], focal adhesion kinase [42, 67], and glycogen synthase kinase 3 (GSK3; [43, 48]. Normalization of spine parameters in CAP1-KO neurons upon expression of a CAP1 variant with a mutated proline-rich motif (CAP1-P1) suggested that its proline-rich domain and, hence, its interaction with profilin were not relevant in spines. Nevertheless, it will be exciting to test in future studies whether other proteins interact with or regulate CAP1 in spines and whether these proteins control CAP1-cofilin1 interaction and synaptic actin dynamics. This will also include post-translational modifications of CAP1, since its phosphorylation by various kinases including GSK3 reportedly modulated binding of cofilin1 and actin [75, 76].

Apart from CAP1, mammals express a second family member, CAP2, with restricted expression pattern and abundance in striated muscles and brain [58, 59]. Similar to CAP1, CAP2 is expressed in the postnatal brain and located in spine heads from cortical and hippocampal neurons [31, 49]. However, while studies in mutant mice established important CAP2 functions in heart physiology and skeletal muscle development [9, 12, 28, 47], neuronal CAP2 functions are less clear. Increased spine density has been reported for cerebral cortex neurons lacking CAP2, but this study unfortunately lacked a detailed spine morphometric analysis and did not provide mechanistic insights [31]. Conversely, spine density was unchanged upon shRNA-mediated CAP2 knockdown (CAP2-KD) in hippocampal neurons, and spine length and width were both slightly increased [49]. However, compared to roughly 30% increased spine size in CAP1-KO neurons, the effect of CAP2-KD on spine morphology was rather mild, suggesting that CAP1 is the key family member in spines. Interestingly, this study revealed CAP2-mediated cofilin1 recruitment into spines upon induction of long-term potentiation (LTP), which depended on disulfide bond-mediated CAP2 dimerization [49]. Remarkably, CAP2-dependent recruitment of cofilin1 into spines was required for LTP-triggered spine remodeling and potentiation of synaptic transmission [49]. Although spine changes in CAP2-KD neurons suggested a role for CAP2 in synaptic actin dynamics, this has not been directly tested in this study. Moreover, it remained to be tested whether CAP2 and cofilin1 cooperate in regulating spine morphology in basal conditions and whether CAP2 was essential for synaptic cofilin1 activity as both shown in the present study for CAP1. Furthermore, it needs to be tested whether CAP1 and its interaction with cofilin1 are relevant for spine morphological changes associated with synaptic plasticity. Nevertheless, a model in which both CAP1 and CAP2 cooperate with cofilin1 in synaptic actin dynamics, spine morphology, and structural plasticity is very appealing, and it will be exciting in future studies to dissect CAP1- vs CAP2-specific mechanisms and to test whether or not CAP1 and CAP2 cooperate and/or are functionally redundant in spines.

In summary, we here identified CAP1 as an essential novel actin regulator in excitatory synapses that is relevant for organization and dynamics of postsynaptic F-actin and thereby controls spine density and morphology. Mechanistically, our data revealed mutual functional dependence of CAP1 and cofilin1 in spine morphology, thereby unravelling a novel synaptic actin regulatory mechanism. Our data let us hypothesize that CAP1 is equally important as cofilin1 for brain function and behavior, and that CAP1 dysregulation may contribute to the pathologies of neuropsychiatric disorders as it has been shown for cofilin1 [10, 78].

## Materials and methods

### Transgenic mice

Mice were housed in the animal facility of the University of Marburg on 12-h dark–light cycles with food and water available ad libitum. Treatment of mice was in accordance with the German law for conducting animal experiments and followed the guidelines for the care and use of laboratory animals of the U.S. National Institutes of Health. Killing of mice has been approved by internal animal welfare authorities. Generation of CAP1^flx/flx^ and Cfl1^flx/flx^ mice has been described before [2, 61]. For our analysis, neurons of three different mouse strains have been used: CAP1^flx/flx^, Cfl1^flx/flx^, and CAP1^flx/flx^/Cfl1^flx/flx^. Neurons transfected with catalytically inactive mCherry-Cre (Cre-mut) have been used as controls. Transfection of catalytically active mCherry-Cre (Cre) caused recombination of floxed alleles and thereby inactivation of CAP1 and/or cofilin1. Neurons from Nestin-Cre-mediated, brain-specific CAP1-KO mice have been exploited for validating specificity of CAP1 antibodies. Generation of these mice has been described before [61].

### Cell culture and transfection

Primary hippocampal neurons from embryonic day 18.5 (E18.5) mice were prepared as previously described [64]. Briefly, hippocampi were dissociated, and neurons were plated at a density of 62,000/cm^2^ on 0.1 mg/ml poly-l-lysine-coated coverslips. Neurons were cultured in Neurobasal medium containing 2% B27, 1 mM GlutaMax, 100 µg/ml streptomycin, and 100 U/ml penicillin (Gibco, Thermo Fisher) in a humidified incubator at 37 °C with 5% CO_2_. Neurons were transfected at DIV6 with a total amount of 1 µg plasmid/well of 24-well plates using Lipofectamine 2000 reagent (Thermo Fisher) according to manufacturer’s protocol. In all experiments, the same amount of each individual construct (see below) has been transfected. Empty pcDNA3.1 vector has been added to set total DNA amount to the desired quantity.

HT-22 cells were plated at a density of 10,000 cells/cm^2^ in cell culture dishes. 24–30 h after plating, HT-22 cells were transfected with 20 µg plasmid/dish using Lipofectamine 2000 reagent (Thermo Fisher). Cell culture medium was changed once roughly 20 h after transfection. 40–48 h later, HT-22 cells were harvested by scraping and homogenized in 1500 µl lysis buffer (50 mM Tris/HCl pH 7.5, 150 mM NaCl, 1% Triton-X, Roche Proteinase inhibitors) using a dounce homogenizer. HT-22 lysates were then left on ice for approximately 30 min before centrifugation. The supernatant of each condition was collected and used for further analysis.

CAP1-eGFP overexpression plasmid-pcDNA3.1-CAP1-eGFP was purchased from Genscript. Other overexpression constructs, such as pEGFP-C1-CAP1, pmCherry-C1-CAP1, and pCMV-Myc-N-CAP1, were generated by amplification of CAP1 ORF from pcDNA3.1-CAP1-eGFP plasmid and cloning it in frame between corresponding restriction sites. Point mutations were introduced according to modified site-directed mutagenesis protocol [34] by exploiting restriction sites and oligonucleotides as listed in Table S7. In all analyses, only neurons that have taken up all transfected constructs have been analyzed, which has been checked for each individual neuron by visual inspection at the microscope. This included myc antibody staining of all neurons transfected with myc-tagged constructs.

### Immunocytochemistry

Neurons were washed once with phosphate-buffered saline (PBS), fixed in 4% paraformaldehyde (PFA)/sucrose in PBS for 15 min, and rinsed in PBS three times. After 10 min incubation in carrier solution (CS; 0.1% gelatin, 0.3% Triton-X100 in PBS), neurons were incubated with primary antibodies in CS for 2 h. Thereafter, neurons were washed with PBS three times for 5 min and incubated with secondary antibodies in CS for 45 min. After washing five times with PBS, coverslips were mounted onto microscopy slides using AquaPoly/mount (Polysciences Inc.). Second last washing step included Hoechst if used.

For STED microscopy, neurons were incubated with permeabilization buffer (0.2% Triton-X-100 in PBS) for 10 min after fixation and washing with PBS. Subsequently, neurons were washed twice with PBS for 5 min and incubated with blocking buffer (BB, 10% horse serum with 0.1% Triton-X-100 in PBS) for 45–60 min. Thereafter, neurons were incubated with primary antibodies in BB o/n at 4 °C. After three 10 min PBS washing steps, neurons were incubated with secondary antibodies in BB for 1 h and washed with PBS five times for 10 min. In case phalloidin was included, neurons were incubated with phalloidin-Atto647N in PBS o/n at 4 °C. Coverslips were mounted onto microscopy slides with Mowiol-488 mounting medium (ROTH, prepared according to manufacturer’s instructions). Tables S8–9 provide lists of primary and secondary antibodies, respectively, including Hoechst and phalloidin.

### Dendrite morphology

For the analysis of dendrite morphology, only neurons expressing the volume marker GFP have been exploited. Images were acquired with Zeiss LSM 5 PASCAL and PASCAL LSM5 software from a single optical plane with a 20 × objective at a resolution of 1024 × 1024 pixels. Background of images was removed using the ImageJ extension program FIJI (https://fiji.sc). Dendrite morphology was assessed by using two different types of analyses. Sholl analysis was done with the FIJI Sholl Plugin. The center of the cell body was marked manually and the plugin was run with the following settings: start radius: 0 µm, step size: 10 µm, end radius: NaN, and samples per radius: 1. More detailed analysis was carried out with the program NeuroMath (version 3.4.8; [54]. Settings were applied as follows: noise level: 1, measure type: cell morphology, segmentation type: threshold, min. cell intensity: 50, min. area: 100, max. area: 600, min. diameter: 5, max. axial ratio: 10, and min. neurite length: 32. Neurons of three independent biological replicates, each with eight images per condition, were analyzed.

### Spine analysis

For the analyses of spine density and morphology, neurons expressing either GFP or dsRed, which both served as volume markers, have been exploited. For each analysis, neurons of all groups expressed the same volume marker. Images were acquired with Leica TCS SP5 II LSM and LAS AF software using a 63 × oil immersion objective. Images were acquired with a resolution of 2048 × 2048 or 1024 × 1024 pixels as z-stacks of 9 optical planes with a step size of 0.49 μm and projected to a single-plane image (maximum projection). To quantify spine density, individual spines were tagged with a circle of 2.254 µm^2^ (FIJI ‘oval’ tool). Spine number was normalized to the length of the analyzed dendrite determined by using ‘freehand line’ tool. Spine volume was determined simultaneously by measuring mean signal intensity in the aforementioned circle. Usually 250–300 spines were counted per neuron from secondary and tertiary basal dendrites. For every neuron, the mean signal intensity of all analyzed spines was calculated and normalized to mean signal intensity of the respective dendritic shafts. Neurons of three independent biological replicates were analyzed, each with 5 neurons per condition. Spine morphology (total length, spine head length, and width) was analyzed by using FIJI ‘freehand selection’ tool in images of dendrite sections with a length of approximately 30 µm. Based on these values, spines were categorized as shown in scheme in Fig. S3D. Spines that did not fit into these categories were classified as ‘other.’ The number of spines was normalized to the length of the respective dendrite. Neurons of three independent biological replicates were analyzed, each with five neurons per condition. Intensity profiles in confocal images were acquired with FIJI ‘plot profile’ tool. Lines were selected in a way that they cover two spines and interjacent dendritic shaft. GFP ratios between spine head vs dendritic shaft or vs spine neck were analyzed with ‘freehand selection’ tool. Like this, mean signal intensities in spine head, spine neck, or an underlying piece of dendrite were determined, which were used to calculate the ratios of mean signal intensities.

### FRAP analysis

Hippocampal neurons were cultured on 35 mm glass bottom dishes (WillCo-dish®) coated with poly-l lysine, transfected at DIV7 with pEGFP-C1-Actin and pCig2-CRE-mCherry or pCig2-CRE-mut-mCherry plasmids. The FRAP imaging was carried out with Leica TCS SP5 II (FRAP wizard) equipped with a temperature-controlled chamber. DIV16-17 neurons were imaged with 63 × objective at 35 °C in 1 × HBSS (Gibco; supplemented with 4 mM NaCO_3_ and 2 mM CaCl_2_). The following imaging settings were applied: format 512 × 512 pixel, speed 700 Hz, 2-line averaging, pinhole 300 µm, 15% of argon laser power, bleaching with 100%, and image acquisition with 3–7% power intensity of AOTF 488 nM (FRAP wizard). Imaging/bleaching program: prebleaching 5 × 2 s, bleaching 5 × 1.5 s (3 µm diameter), and postbleaching 20 × 2 s, 10 × 5 s and 20 × 10 s.

The image series were analyzed using FIJI as previously reported [39]. Briefly, background and bleaching correction was applied and normalized fluorescence intensity for each time point was calculated. Nonlinear curve fitting (one phase exponential association) of the fluorescence intensity was performed with GraphPad Prism, where the net recovery after photobleaching is provided by the following equation: *Y* = *Y*0 + (Plateau − *Y*0) × (1 − exp(− *K* × *x*)), where *Y*0 is the *Y* value when time is zero directly after the bleaching impulse and Plateau is the *Y* value at infinite times, expressed as a fraction of the fluorescence before bleaching and was used to determine the dynamic actin pool (F-actin dynamic). The stable pool (F-actin stable) is the fraction of fluorescence that does not recover within the imaging period of 300 s calculated as 1-(F-actin dynamic), *K* is the rate constant, and *T* is the time constant, expressed in s; it is computed as the reciprocal of K.

### STED microscopy and image analysis

STED nanoscopy was performed using a Leica SP8-3xSTED and an Abberior Facility line imaging system. The Leica SP8-3 × STED microscope was used for the acquisition of three color STED images. A white light laser was used for excitation at the wavelengths 488 nm for Alexa-488, 580 nm for Abberior-STAR-580, and 650 nm for Atto-647N. Fluorophore depletion was achieved with a 775 nm laser for AttoFluor-647N/Abberior-STAR-580 and a 592 nm laser for Alexa-488. All images were acquired using a 100 × oil objective (Leica, HC APO CS2). Emission light was detected in bins: 660–730 nm for Atto-647N, 590–620 nm for Abberior-STAR-580, and 500–530 nm for Alexa-488. Gated detection was applied with a delay of 0.3–1.5 ns. Pinhole size was set to 1 AU. Images were acquired with a 5 × zoom resulting in a pixel size of 22.73 nm, 1024 by 1024 and a 16 × line averaging.

Additional STED imaging was performed on an Abberior STED Facility line microscope (Abberior Instruments GmbH) with an UPLSAPO 100 × oil immersion objective lens (NA 1.4). Pixel size was set to 20 nm for all images. Images were obtained from a 16 × frame accumulation. Excitation was achieved with pulsed diode lasers PDL-T 488, 561 and 640. Both the red and far-red channel were depleted using a 775 nm laser (PFL-40-3000-775-B1R). Pinhole size was set to 1AU. Gated detection was applied for both channels.

Postsynaptic distribution of CAP1 was analyzed in mushroom-like spines of two independent DIV16 hippocampal cultures using a custom-written script in MATLAB (R2015a, MathWorks, Inc) that has been described previously [41]. For analysis, phalloidin (Fig. 1J–L) or GFP (Fig. 3F–H) signals were slightly oversaturated to facilitate outlining the spine head. Postsynaptic distribution of phalloidin, Shank3, PSD-95, and Homer in mushroom-like spines from CTR and CAP1-KO neurons has been analyzed in three independent DIV16 hippocampal cultures. Line scan analysis was carried out with FIJI ‘plot profile’ tool (line width:15).

### Proximity ligation assay

Rat hippocampal neuronal primary cultures were prepared from embryonic day 19 (E19) rat hippocampi as previously described [50]. Primary hippocampal cultures were transfected with GFP at DIV9-10 and fixed at DIV15 with 4% PFA/sucrose in PBS for 10 min at room temperature (RT), then washed three times with PBS. Neurons were permeabilized with 0.1% Triton X-100 in PBS for 15 min at RT. After incubation with the blocking solution of the PLA kit (Duolink® PLA Technology), cells were incubated o/n at 4 °C with primary antibodies against CAP1 (1:100, Abnova, H00010487-M02) and cofilin1 (1:100, Cell Signaling, 5175). According to the manufacturer’s instructions, after two washes with Wash Buffer A, secondary antibodies conjugated with oligonucleotides were added and the oligonucleotides of the bound probes were ligated and amplified by a fluorescent polymerase that visualizes the PLA clusters. Coverslips were then washed three times with decreasing concentration of Buffer Solution B. After this, cells were labeled with primary antibody against GFP (1:500, Millipore, AB16901) for 1 h at RT, washed, and then incubated with secondary antibody. After three washes with PBS, coverslips were mounted on slides in Fluoromount™ Aqueous Mounting Medium (Sigma-Aldrich).

### Protein extraction and synaptosomes

Prefrontal cortices were snap frozen in liquid N_2_ and stored at -80 °C. Homogenization was done with 6–10 strokes in 750 µl lysis buffer (50 mM Tris-pH 7.5, 150 mM NaCl, 1% Triton-X100, 1 × Complete Protease Inhibitor Cocktail, Roche) using a dounce homogenizer. After 20 min centrifugation, 100 µl supernatant was collected and used for further analysis. Protein lysates from isolated neurons were generated from five coverslips (250,000 neurons/coverslip) by treating with 50 µl lysis buffer incl. PST (1 × PhosSTOP, Roche) on ice. After 10 min incubation on the shaker at 4 °C, neurons were lysed by pipetting 10 times up and down.

Synaptosomes were purified as described [35]. Briefly, both cerebral cortices and both hippocampi of a P20 mouse were homogenized in chilled sucrose solution (0.32 M sucrose, 1 mM EDTA, 1 mg/ml bovine serum albumin, 5 mM HEPES, pH 7.4) with 8 strokes using a dounce homogenizer. After removing nuclei and cell debris by centrifugation (3000*g*, 10 min, 4 °C), supernatant was further pelleted at 14,000*g* for 10 min at 4 °C. A pellet containing synaptosomes was enriched on a floatation gradient containing 45% Percoll in Krebs–Ringer solution (140 mM NaCl, 5 mM KCl, 25 mM HEPES, 1 mM EDTA, 10 mM glucose, pH 7.4), and the supernatant (S2) containing microsomes and soluble enzymes was stored at −80 °C. The synaptosomal fraction was resuspended in Krebs-HEPES solution (124 mM NaCl, 3 mM KCl, 1 mM MgCl_2_, 2 mM CaCl_2_, and 10 mM glucose buffered with 25 mM HEPES, pH 7.4) for subsequent use. For solubilization, synaptosomes were diluted 1:1 with ice-cold 0.1 mM CaCl_2_; then an equal volume of 2 × solubilization buffer (2% TX-100, 0.2 mM CaCl_2_, 40 mM Tris buffer, pH 7.4) was added. Soluble or membrane-associated synaptic proteins were separated by centrifugation at 10,000*g* for 30 min at 4 °C. Thereafter, insoluble protein fraction was resuspended in lysis buffer (5% SDS, 5 mM EDTA, 140 mM NaCl, 50 mM Tris buffer, pH 8.0) with the same volume as the soluble fraction. Hence, compared to synaptosomal lysates, soluble and insoluble fractions were diluted 1:4.

Proteins were separated on a 10% SDS-PAGE and transferred o/n at 4 °C and 27 V onto a polyvinylidene difluoride membrane (GE Healthcare) using Mini-Protean electrophoresis system (Biorad). Non-specific antibody binding was blocked in Tris-buffered saline containing 5% milk powder and 0.2% Tween 20 (TBS-T/milk). Membranes were incubated with primary antibody diluted in TBS-T/milk dilutions for 2 h at RT. Thereafter, membranes were washed three with TBS-T/milk and incubated with horseradish peroxidase-conjugated secondary antibodies (Thermo Fisher Scientific) in TBS-T/milk for 1 h. Membranes were washed three times with TBS-T before developing with Amersham ECLplus reagent (GE Healthcare). Tables S8–9 include lists of antibodies.

### Co-immunoprecipitation with hippocampal homogenates

Mouse hippocampi were homogenized at 4 °C in an ice-cold buffer with Roche Complete™ Protease Inhibitor Cocktail, phosphatase inhibitors (PhosSTOP™, Sigma-Aldrich), 0.32 M Sucrose, 1 mM HEPES, 1 mM NaF, 0.1 mM PMSF, and 1 mM MgCl_2_ using a glass–teflon homogenizer. Aliquots of 50 μg of homogenate obtained from mouse hippocampus were incubated for 1 h at 4 °C in 200 μl of RIPA buffer (200 mM NaCl, 10 mM EDTA, 10 mM Na_2_HPO_4_, 0.5% NP-40, 0.1% SDS) and SureBeads Protein A magnetic beads (Bio-Rad) as pre-cleaning procedure. The supernatant was incubated with primary antibody against CAP1 (Abnova, H00010487-M02) o/n at 4 °C. SureBeads Protein A magnetic beads were added and incubation was continued for 2 h at RT. After three washes with RIPA buffer, beads were resuspended in sample buffer and heated for 10 min. Beads were collected by centrifugation and all supernatants were applied onto SDS-PAGE. The precipitated immunocomplex was analyzed by anti-cofilin1 (Cell Signaling, #5175), anti-CAP1 (Proteintech, 16231-1-AP) and anti-β2 adaptin antibody (BD Biosciences, 610381).

### Co-immunoprecipitation in HT-22 cells

For each CoIP assay 50 µL Dynabeads™ Protein G (Invitrogen, #10003D) was used and incubated with 2 µg anti-GFP antibody (Invitrogen, G10362) for 1.5 h on a rotating platform. In the meantime, transfected HT-22 cells were lysed and homogenized and supernatant was obtained after centrifugation. After incubation, magnetic beads were washed three times with CoIP wash buffer (20 mM Tris/HCl pH 8.0, 100 mM NaCl). 600 µl of supernatant for each condition was applied and samples were left for incubation for approximately 2 h at 4 °C on a rotating platform. Subsequently beads were washed two times with CoIP wash buffer before resuspension in 120 µl of sample buffer. For validation samples and controls were heated for 5 min and applied onto SDS-PAGE. Afterward, proteins were blotted onto a nitrocellulose membrane before incubation with a c-myc monoclonal antibody (Invitrogen, MA1-980). Expression levels of GFP and GFP-cofilin in the inputs were determined by exploiting an anti-GFP antibody (Abcam, ab13970). Immunoblots were analyzed by using the Odyssey DLx imager (Li-Cor).

### Statistical analysis

Values are reported as mean ± standard error of the mean (SEM) and (if not otherwise stated) based on three independent biological replicates. For data with single comparison statistical significance was calculated using Student’s *t*-test (two-sample, unpaired), comparison of spine type distribution was tested with *χ*^2^-test and rescue experiments were tested for statistical significance by one-way ANOVA followed by Dunnett’s post hoc test. In all experiments, experimenters were blind to the genotype during image acquisition and analysis.

### Supplementary Information

Below is the link to the electronic supplementary material.

Figure S1. (**A**) Graph showing head-shaft ratio of GFP divided by head-shaft ratio of dsRed in thin and mushroom-like spines from neurons transfected with dsRed together with either GFP or CAP1-GFP. (**B**) Dendritic shaft of a hippocampal neuron expressing CAP1-GFP (green) and the F-actin marker mCherry-LifeAct (red). (**C**) Fluorescence intensity profiles along white line shown in Fig. S1B. Left-to-right direction in graph corresponds to top-to-bottom direction in micrograph. (**D**) Antibody staining against endogenous CAP1 (green) in a hippocampal neuron expressing dsRed (red). (E) Antibody staining against CAP1 (green) and doublecortin (Dcx, red) in hippocampal neurons from CAP1flx/flx mice (CTR) and brain-specific CAP1-KO mice (Schneider, 2021a). Neurons were additionally stained with the DNA dye Hoechst (blue). White boxes indicate areas shown at higher magnification. Scale bars (µm): 2 (B, D), 20 (E). ***:* P*<0.001. Supplementary file1 (JPG 6036 KB)

Figure S2. STED images of hippocampal neurons stained with antibodies against (**A**) Shank3 (magenta) and Bassoon (cyan) and (**B**) CAP1 (shown in ‘fire’). Boxes indicate synapses shown at higher magnification in Figs. 1H or S2C. (**C**) High magnification of excitatory synapses shown in Fig. S2A-B. Shank3 immunoreactivity is shown in magenta, Bassoon in cyan and CAP1 in ‘fire’ (single channel) or green (merge). (**D**) Integrated fluorescence intensity profiles along transparent boxes shown in Fig. S2C, direction is indicated by dashed arrows. Scale bars (µm): 2 (A), 1 (C). Supplementary file2 (JPG 10731 KB)

Figure S3. STED images of hippocampal neurons stained with (**A**) phalloidin (cyan) and with antibodies against Shank3 (magenta) and (**B**) CAP1 (shown in ‘fire’). Boxes indicate dendritic spines shown at higher magnification in Figs. 1J or S3C. (**C**) High magnification of dendritic spines shown in Fig. S3A-B. Phalloidin is shown in ‘red hot’ (single channel) or cyan (merge), Shank3 in magenta and CAP1 in ‘fire’ (single channel) or green (merge). Scale bars (µm): 2 (A), 1 (C). Supplementary file3 (JPG 12979 KB)

Figure S4. Micrographs of CAP1^flx/flx^ neurons transfected with either catalytically inactive mCherry-Cre mutant (red, left) or catalytically active mCherry-Cre (red, right). Neurons were co-transfected with GFP (green) and stained with an antibody against CAP1 (magenta) and the nuclear marker Hoechst (cyan). GFP was used to outline transfected neurons (dashed white line). Boxes indicate areas shown at higher magnification. Scale bar (µm): 20 (low magnification), 10 (high magnification). Supplementary file4 (JPG 10365 KB)

Figure S5. (**A**) Micrographs of GFP-expressing CTR and CAP1-KO neurons that were used for morphometric analysis. Graphs showing (**B**) number of primary neurites, (**C**) number of branching points, and (**D**) branching points normalized to dendritic length. (**E**) Black/white images of neurons shown in Fig. S5A that were used for Sholl analysis. Intersections of dendritic shafts and concentric circles with increasing radii (10 µm increments) are indicated by colored dots. Graphs showing (**F**) number of intersections at each radius, (**G**) maximal number of intersections, (**H**) radius with highest count of intersections and (**I**) ramification index (maximal intersections/primary neurites) for CTR and CAP1-KO neurons. Grey box in F indicates radii, in which the number of intersections were different between both groups. Scale bars (µm): 100 (A, E). ns:* P*≥0.05, *:* P*<0.05. Supplementary file5 (JPG 3209 KB)

Figure S6. (**A**) Scheme indicating morphometric parameters used for spine categorization (Hering, 2001). Graphs showing (**B**) length and width of filopodia-like spines, (**C**) total length, head length and head width of thin spines as well as (**D**) length and width of stubby spines in CTR and CAP1-KO neurons. ns:* P*≥0.05. Supplementary file6 (JPG 1596 KB)

Figure S7. Micrographs of dendritic shafts from (**A**) CTR and (**B**) CAP1-KO neurons. Neurons were transfected with GFP (images were acquired by conventional confocal microscopy) and stained with phalloidin and an antibody against Shank3 (images of both acquired by STED nanoscopy). White boxes indicate dendritic spines shown at higher magnification in Fig. S7C-D. Yellow boxes indicate areas shown at higher magnification in insets, in which periodic actin rings were visible. High magnification of dendritic spines in (**C**) CTR and (**D**) CAP1-KO neurons. GFP is shown in grayscale (single channel) or green (merge), phalloidin in ‘red hot’ (single channel) or cyan (merge) and Shank3 in grayscale (single channel) or magenta (merge). Scale bars (µm): 2 (A, B), 1 (C, D). Supplementary file7 (JPG 9147 KB)

Figure S8. Micrographs of dendritic spines from GFP-transfected (green) (**A**) CTR and (**B**) CAP1-KO neurons stained with antibodies against Homer (magenta) and PSD-95 (cyan). Scale bars (µm): 1 (A, B).Supplementary file8 (JPG 8761 KB)

Figure S9. Micrographs of DIV16 CAP1flx/flx neurons triple transfected at DIV6 with GFP (green), mCherry-Cre (red) and myc-WT-CAP1 (visualized by myc antibody staining (magenta)) demonstrating that neurons expressed all three transfected constructs. Scale bars (µm): 50. Supplementary file9 (JPG 2384 KB)

Figure S10. (**A**) Immunoblots showing expression of CAP1 and cofilin1 at various postnatal stages in cerebral cortex. GAPDH was used as loading control and for normalization to protein load in statistical analyses. Graph showing expression levels of (**B**) CAP1 and (**C**) cofilin1 normalized to postnatal day 1 (P1). Supplementary file10 (JPG 816 KB)

Figure S11. Graphs showing (**A**) length and width of filopodia-like spines, (**B**) total length, head length and head width of thin spines, (**C**) length and width of stubby spines as well as (**D**) total length, head length and head width of mushroom-like spines in CTR and cofilin1-KO neurons. (**E**) Box plots (incl. MV±SEM) showing spine density in CTR and CAP1-KO neurons either expressing GFP or cofilin1-GFP. (**F**) Box plots (incl. MV±SEM) showing spine density in CTR and cofilin1-KO neurons either expressing GFP or CAP1-GFP. ns: P≥0.05, **:* P*<0.01. ***:* P*<0.001. Supplementary file11 (JPG 1375 KB)

Figure S12. Graphs showing (**A**) length and width of filopodia-like spines, (**B**) total length, head length and head width of thin spines, (**C**) length and width of stubby spines as well as (**D**) total length, head length and head width of mushroom-like spines in CTR and dKO neurons. ns:* P*≥0.05, *:* P*<0.05. Supplementary file12 (JPG 1510 KB)

Movie S1. Movie showing FRAP of GFP-actin in a mushroom-like spine from a CTR neuron. Supplementary file13 (AVI 1646 KB)

Movie S2. Movie showing FRAP of GFP-actin in a mushroom-like spine from a CAP1-KO neuron. Supplementary file14 (AVI 1646 KB)

Table S1. Table showing parameters of dendritic morphology in CTR and CAP1-KO neurons as shown in Fig. S5. Significant changes between CTR and CAP1-KO are highlighted by colored font. Supplementary file15 (PDF 73 KB)

Table S2. Table showing spine density and volume in CAP1-KO and cofilin1-KO neurons and their corresponding CTR as shown in Figs. 2B-C and 6B-C. Moreover, the table includes spine density and volume in CTR and CAP1-KO neurons upon expression of myc-tagged CAP1 variants (named in left column) as shown in Fig. 4C-F. Significant changes are highlighted by colored font. Supplementary file16 (PDF 80 KB)

Table S3. Table summarizing morphometric analyses of spines (all spines, spine subtypes) in CAP1-KO, cofilin1-KO and dKO neurons and their corresponding CTR as shown in Figs. 2D-F, 3A, 6D-F, 7D-F, S6B-D, S11A-D and S12A-D. Significant changes are highlighted by colored font.Supplementary file17 (PDF 71 KB)

Table S4. Table summarizing spine type distribution in CAP1-KO, cofilin1-KO and dKO neurons and their corresponding CTR as shown in Figs. 2G, 6G, 7G. Significant changes are highlighted by colored font. Supplementary file18 (PDF 59 KB)

Table S5. Table summarizing spine density and volume in CAP1-KO and cofilin1-KO neurons and their corresponding CTR before/after expression of either GFP-cofilin1 or and GFP-CAP1 (indicated in left column) as shown in Figs. 6J-K, S11E-F. Significant changes are highlighted by colored font. Supplementary file19 (PDF 78 KB)

Table S6. Table summarizing changes in spine density and volume in CTR and dKO neurons before/after expression of CAP1 and cofilin1 constructs named in blue and red column as shown in Fig. 7I-J. Significant changes are highlighted by colored font. Supplementary file20 (PDF 78 KB)

Table S7. List of oligonucleotides used for site-directed mutagenesis on CAP1 constructs. Supplementary file21 (PDF 223 KB)

Table S8. List of primary antibodies used for immunocytochemistry (ICC) and immunoblots (IB). Supplementary file22 (PDF 70 KB)

Table S9. List of secondary antibodies used for immunocytochemistry (ICC) and immunoblots (IB). Supplementary file23 (PDF 324 KB)

## Data Availability

The datasets generated during and/or analyzed during the current study are available from the corresponding author on reasonable request.
