# Correction: Functional interdependence of the actin regulators CAP1 and cofilin1 in control of dendritic spine morphology

**DOI:** 10.1007/s00018-023-05039-5

**Published:** 2024-01-18

**Authors:** Anika Heinze, Cara Schuldt, Sharof Khudayberdiev, Bas van Bommel, Daniela Hacker, Toni G. Schulz, Ramona Stringhi, Elena Marcello, Marina Mikhaylova, Marco B. Rust

**Affiliations:** 1 Molecular Neurobiology Group, Institute of Physiological Chemistry, Philipps-University of Marburg, 35032 Marburg, Germany; 2 Center for Mind, Brain and Behavior (CMBB), University of Marburg and Justus-Liebig-University Giessen, 35032 Marburg, Germany; 3 AG Optobiology, Institute of Biology, Humboldt-University, 10115 Berlin, Germany; 4 Guest Group ‘Neuronal Protein Transport’, Institute for Molecular Neurogenetics, Center for Molecular Neurobiology (ZMNH), University Medical Center Hamburg-Eppendorf (UKE), 20251 Hamburg, Germany; 5 Department of Pharmacological and Biomolecular Sciences, Universita degli Studi di Milano, 20133 Milan, Italy; 6 DFG Research Training Group ‘Membrane Plasticity in Tissue Development and Remodeling’, GRK 2213, Philipps-University of Marburg, 35032 Marburg, Germany; 7 Institute of Chemistry and Biochemistry, Department of Biology, Chemistry and Pharmacy, Freie Universität Berlin, 14195 Berlin, Germany

**Cellular and Molecular Life Sciences (2022) 79:558**

10.1007/s00018-022-04593-8

In this article the statement in the Funding information section was incorrectly given as

‘Open Access funding enabled and organized by Projekt DEAL. This work was supported by research grants from Fondazione Cariplo (2018-0511) and from Deutsche Forschungsgemeinschaft (DFG) RU 1232/7-1 to MBR as well as DFG Emmy Noether Programme MI1923/1-2, DFG RU2419 TP2, DFG CRC1315 Project A3, and Excellence Strategy-EXC-2049-390688087 to MM. Abberior STED System was funded by the DFG-INST 276/760-1.’

and should have read

‘Open Access funding enabled and organized by Projekt DEAL. This work was supported by research grants from Fondazione Cariplo (2018-0511) and from Deutsche Forschungsgemeinschaft (DFG) RU 1232/10-1 to MBR as well as DFG Emmy Noether Programme MI1923/1-2, DFG RU2419 TP2, DFG CRC1315 Project A3, and Excellence Strategy-EXC-2049-390688087 to MM. Abberior STED System was funded by the DFG-INST 276/760-1.’

The original article has been corrected.

