# Supplementary figures and images for: Functional interdependence of the actin regulators CAP1 and cofilin1 in control of dendritic spine morphology
Source: Cell Mol Life Sci. 2022 Oct 20;79(11):558. doi: 10.1007/s00018-022-04593-8 (PMC9585016; doi:10.1007/s00018-022-04593-8)

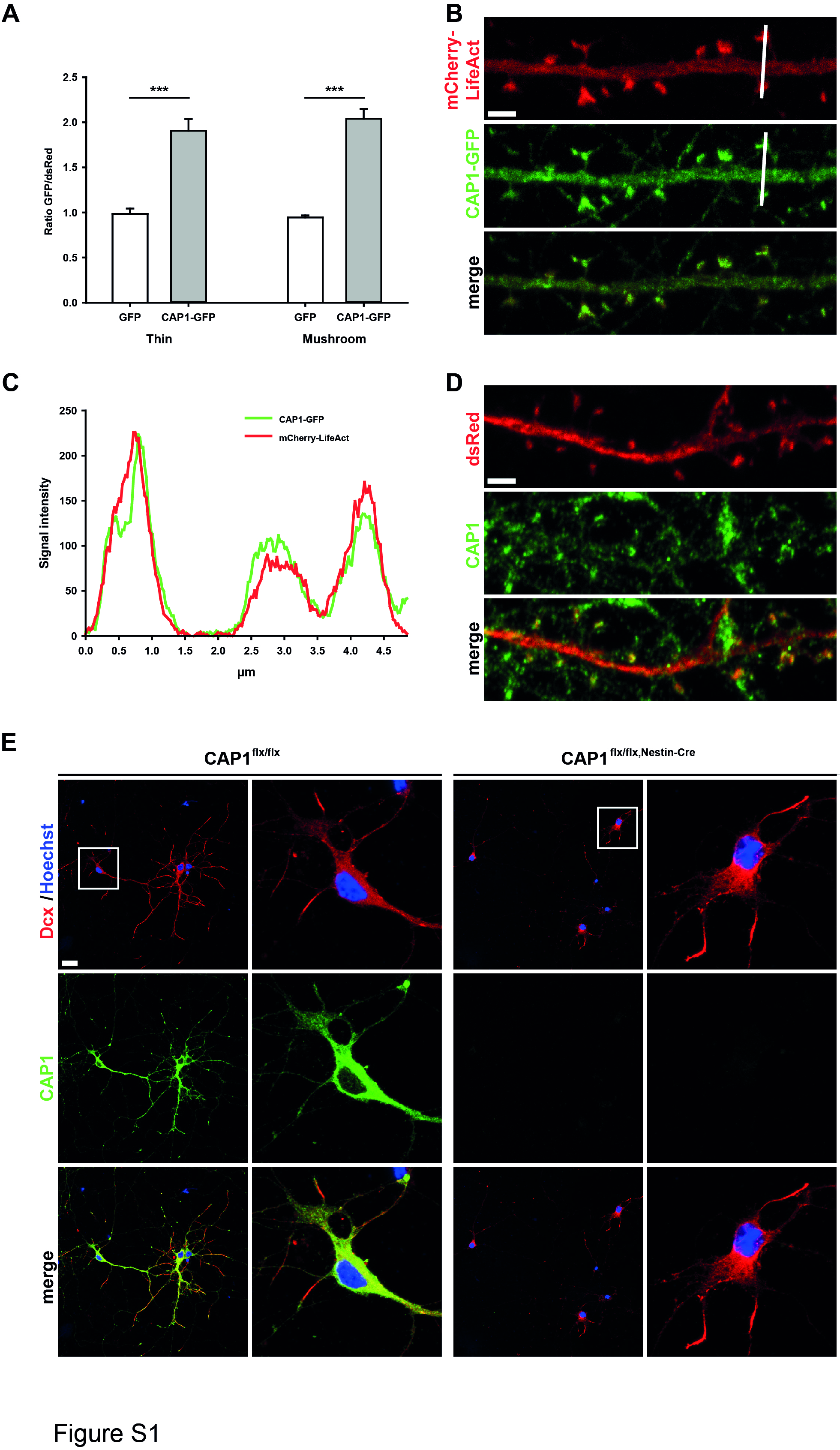

Supplement: Supplementary file 1 — (A) Graph showing head-shaft ratio of GFP divided by head-shaft ratio of dsRed in thin and mushroom-like spines from neurons transfected with dsRed together with either GFP or CAP1-GFP. (B) Dendritic shaft of a hippocampal neuron expressing CAP1-GFP (green) and the F-actin marker mCherry-LifeAct (red). (C) Fluorescence intensity profiles along white line shown in Fig. S1B. Left-to-right direction in graph corresponds to top-to-bottom direction in micrograph. (D) Antibody staining against endogenous CAP1 (green) in a hippocampal neuron expressing dsRed (red). (E) Antibody staining against CAP1 (green) and doublecortin (Dcx, red) in hippocampal neurons from CAP1flx/flx mice (CTR) and brain-specific CAP1-KO mice (Schneider, 2021a). Neurons were additionally stained with the DNA dye Hoechst (blue). White boxes indicate areas shown at higher magnification. Scale bars (µm): 2 (B, D), 20 (E). ***: P<0.001. Supplementary file1 (JPG 6036 KB) [file 18_2022_4593_MOESM1_ESM.jpg]

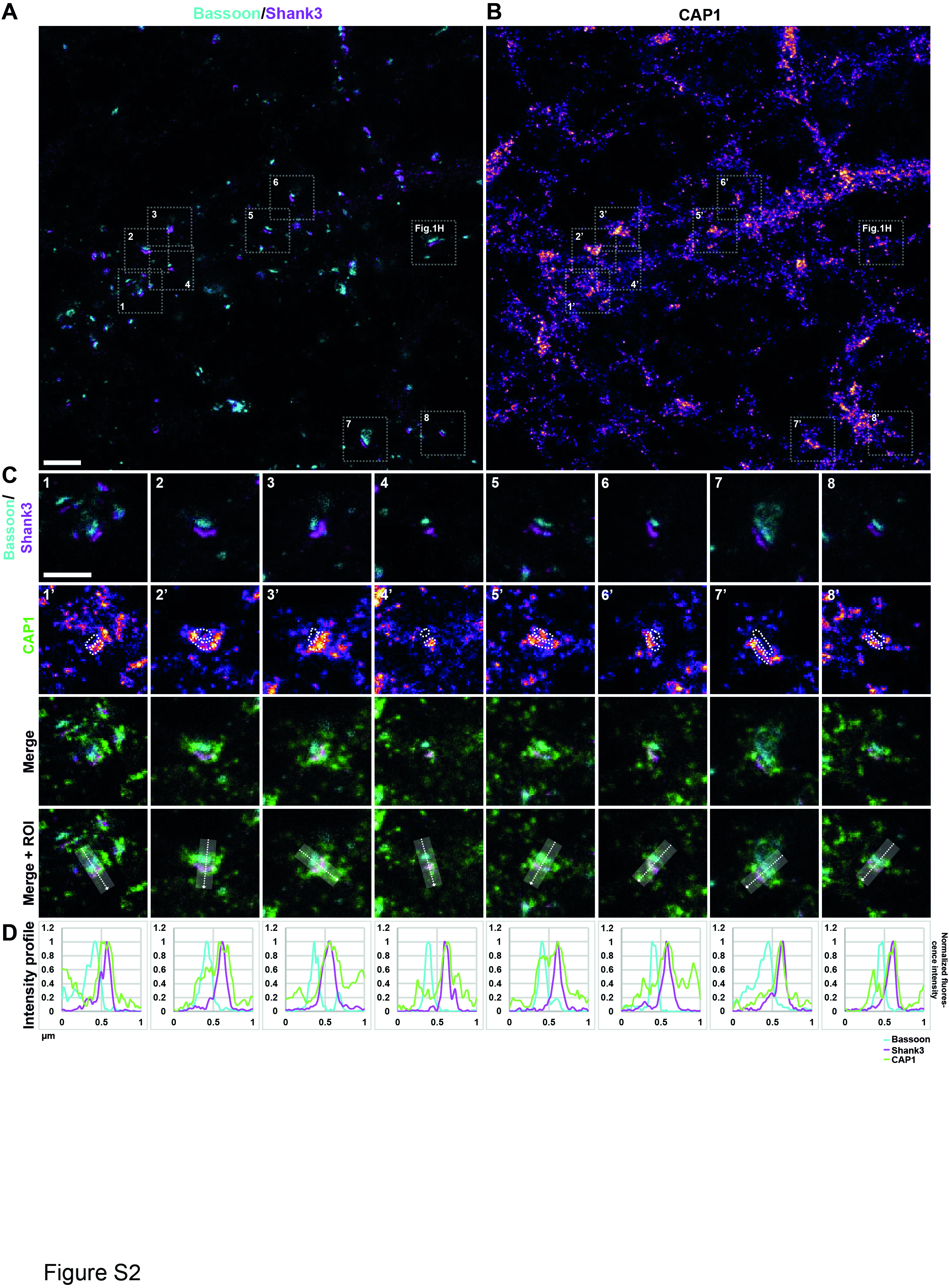

Supplement: Supplementary file 2 — STED images of hippocampal neurons stained with antibodies against (A) Shank3 (magenta) and Bassoon (cyan) and (B) CAP1 (shown in ‘fire’). Boxes indicate synapses shown at higher magnification in Figs. 1H or S2C. (C) High magnification of excitatory synapses shown in Fig. S2A-B. Shank3 immunoreactivity is shown in magenta, Bassoon in cyan and CAP1 in ‘fire’ (single channel) or green (merge). (D) Integrated fluorescence intensity profiles along transparent boxes shown in Fig. S2C, direction is indicated by dashed arrows. Scale bars (µm): 2 (A), 1 (C). Supplementary file2 (JPG 10731 KB) [file 18_2022_4593_MOESM2_ESM.jpg]

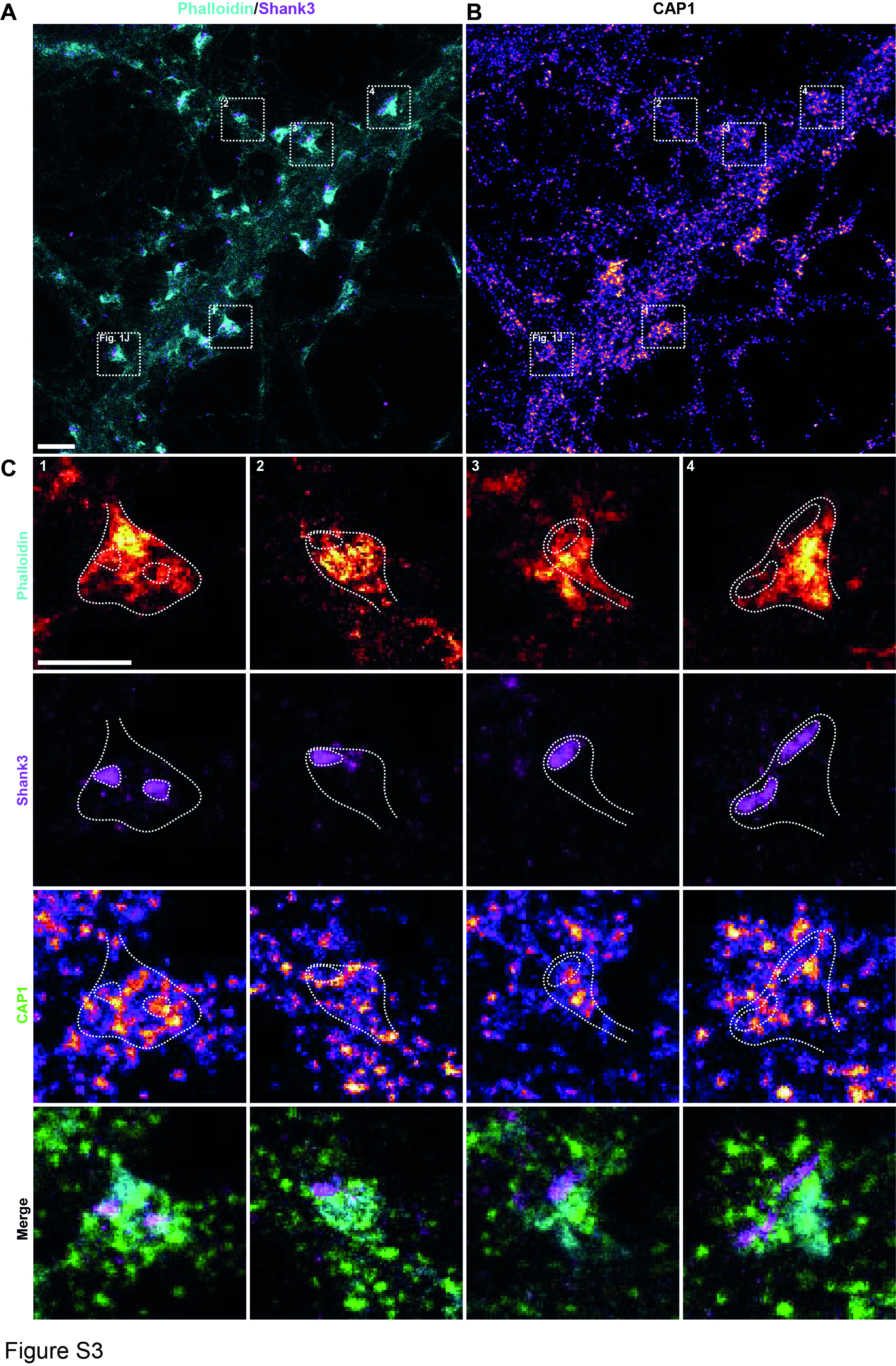

Supplement: Supplementary file 3 — STED images of hippocampal neurons stained with (A) phalloidin (cyan) and with antibodies against Shank3 (magenta) and (B) CAP1 (shown in ‘fire’). Boxes indicate dendritic spines shown at higher magnification in Figs. 1J or S3C. (C) High magnification of dendritic spines shown in Fig. S3A-B. Phalloidin is shown in ‘red hot’ (single channel) or cyan (merge), Shank3 in magenta and CAP1 in ‘fire’ (single channel) or green (merge). Scale bars (µm): 2 (A), 1 (C). Supplementary file3 (JPG 12979 KB) [file 18_2022_4593_MOESM3_ESM.jpg]

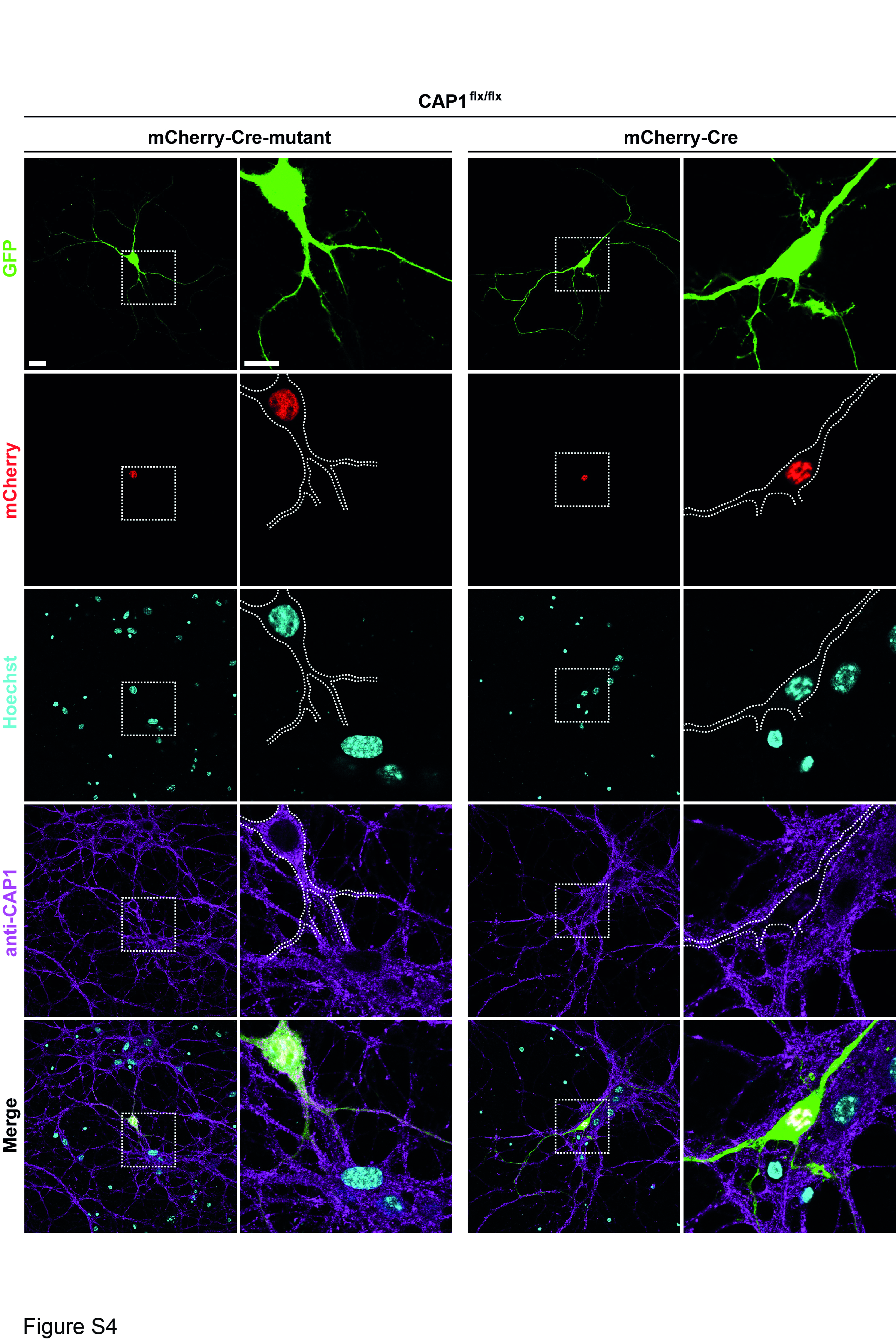

Supplement: Supplementary file 4 — Micrographs of CAP1flx/flx neurons transfected with either catalytically inactive mCherry-Cre mutant (red, left) or catalytically active mCherry-Cre (red, right). Neurons were co-transfected with GFP (green) and stained with an antibody against CAP1 (magenta) and the nuclear marker Hoechst (cyan). GFP was used to outline transfected neurons (dashed white line). Boxes indicate areas shown at higher magnification. Scale bar (µm): 20 (low magnification), 10 (high magnification). Supplementary file4 (JPG 10365 KB) [file 18_2022_4593_MOESM4_ESM.jpg]

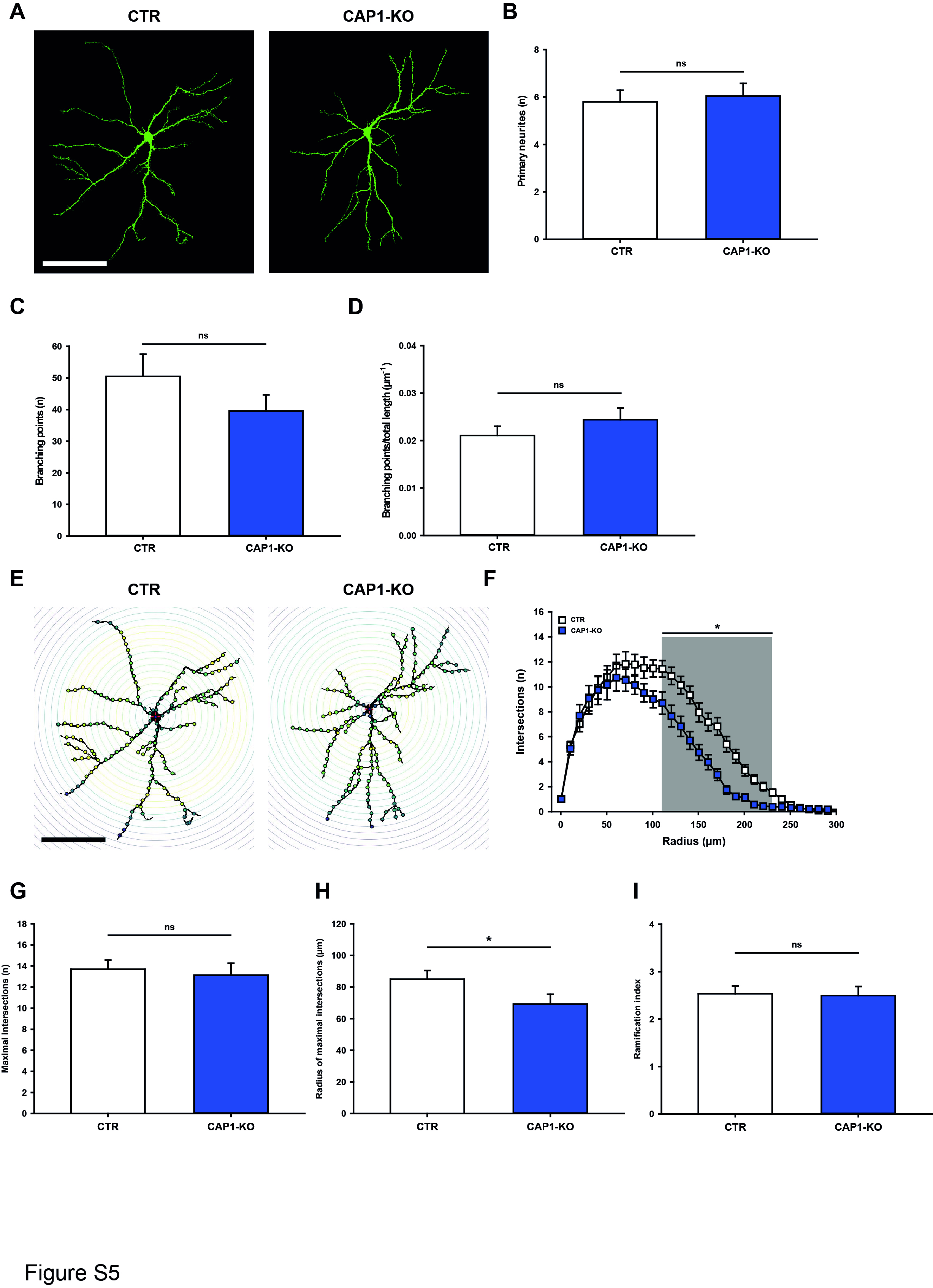

Supplement: Supplementary file 5 — (A) Micrographs of GFP-expressing CTR and CAP1-KO neurons that were used for morphometric analysis. Graphs showing (B) number of primary neurites, (C) number of branching points, and (D) branching points normalized to dendritic length. (E) Black/white images of neurons shown in Fig. S5A that were used for Sholl analysis. Intersections of dendritic shafts and concentric circles with increasing radii (10 µm increments) are indicated by colored dots. Graphs showing (F) number of intersections at each radius, (G) maximal number of intersections, (H) radius with highest count of intersections and (I) ramification index (maximal intersections/primary neurites) for CTR and CAP1-KO neurons. Grey box in F indicates radii, in which the number of intersections were different between both groups. Scale bars (µm): 100 (A, E). ns: P≥0.05, *: P<0.05. Supplementary file5 (JPG 3209 KB) [file 18_2022_4593_MOESM5_ESM.jpg]

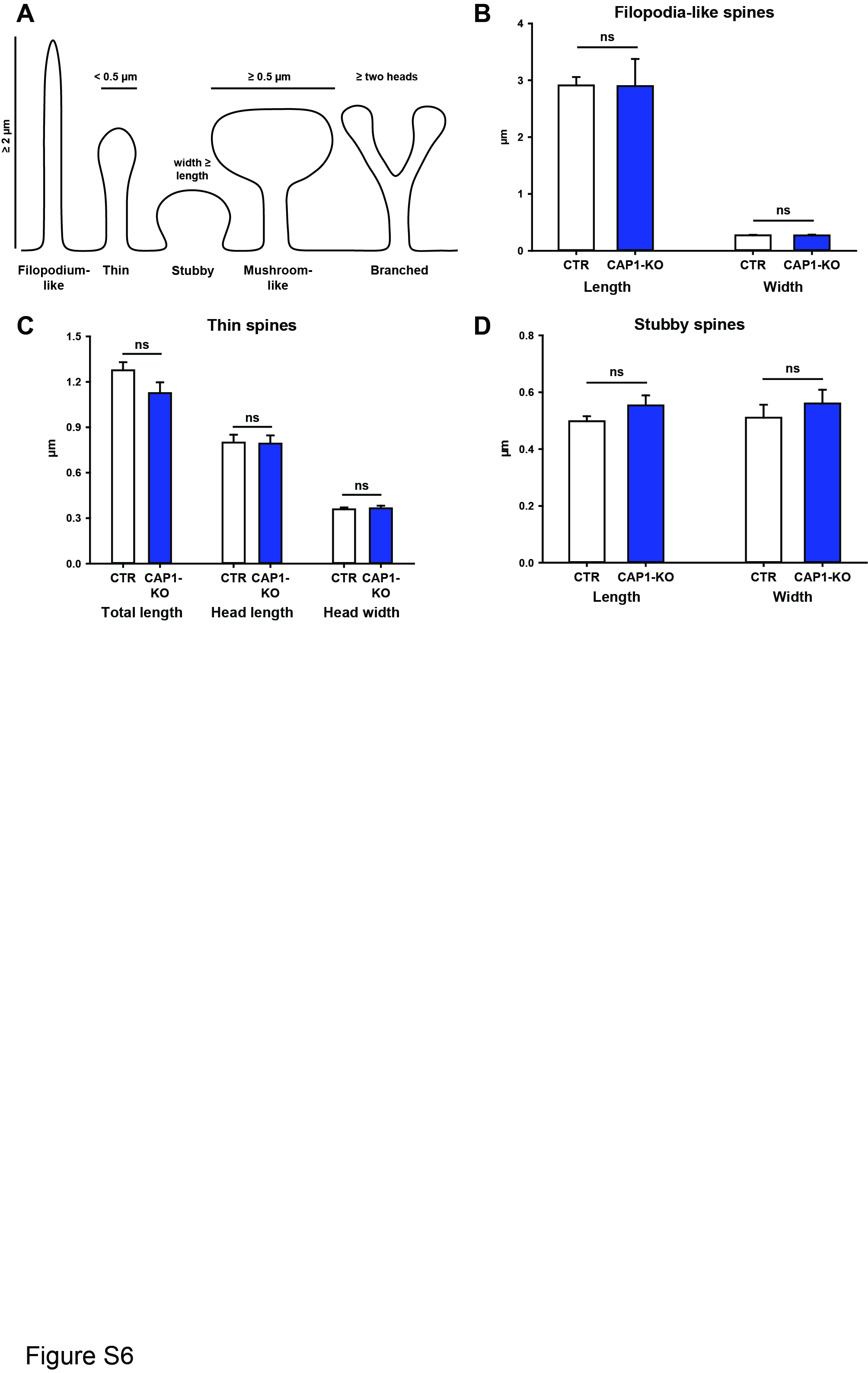

Supplement: Supplementary file 6 — (A) Scheme indicating morphometric parameters used for spine categorization (Hering, 2001). Graphs showing (B) length and width of filopodia-like spines, (C) total length, head length and head width of thin spines as well as (D) length and width of stubby spines in CTR and CAP1-KO neurons. ns: P≥0.05. Supplementary file6 (JPG 1596 KB) [file 18_2022_4593_MOESM6_ESM.jpg]

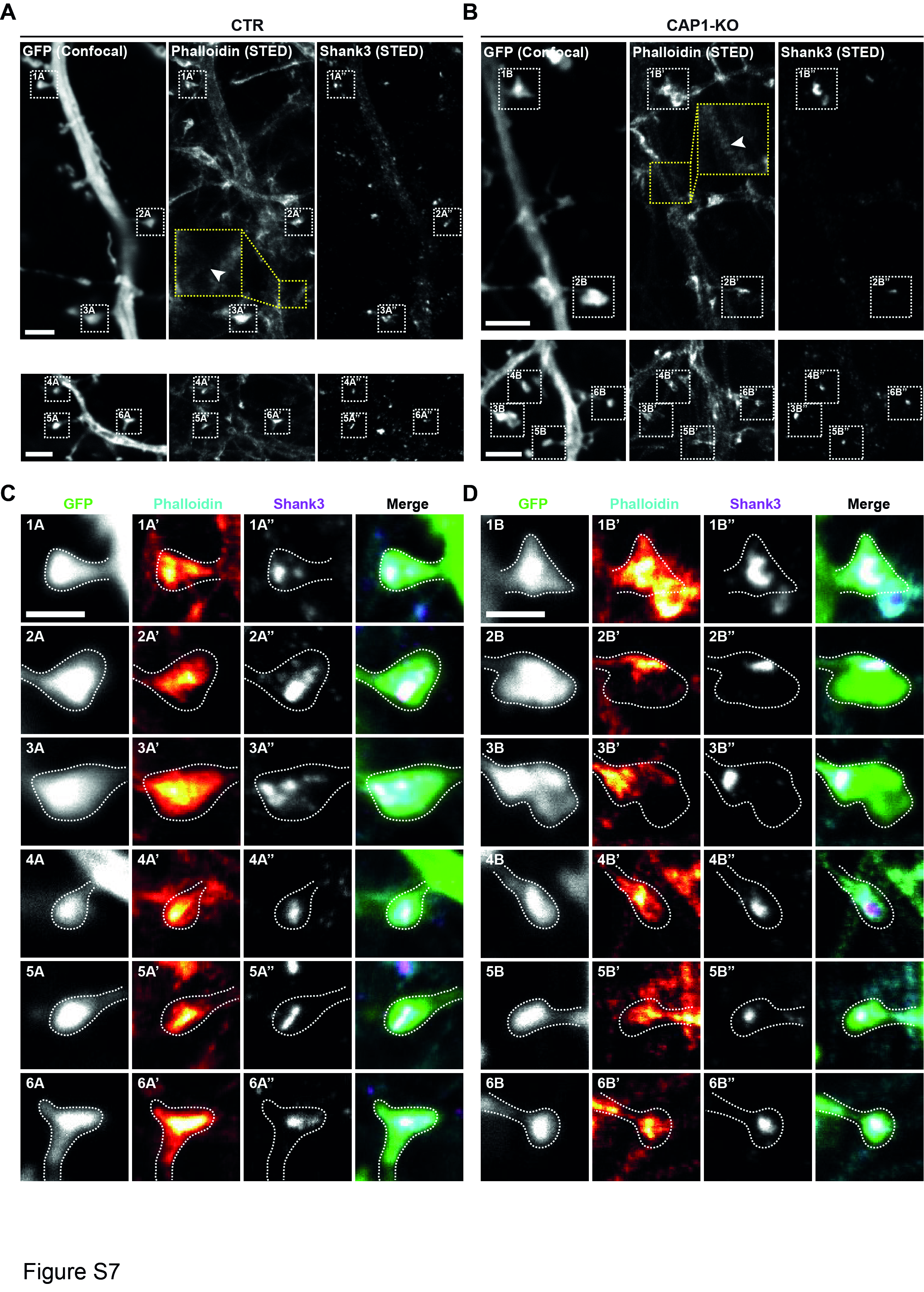

Supplement: Supplementary file 7 — Micrographs of dendritic shafts from (A) CTR and (B) CAP1-KO neurons. Neurons were transfected with GFP (images were acquired by conventional confocal microscopy) and stained with phalloidin and an antibody against Shank3 (images of both acquired by STED nanoscopy). White boxes indicate dendritic spines shown at higher magnification in Fig. S7C-D. Yellow boxes indicate areas shown at higher magnification in insets, in which periodic actin rings were visible. High magnification of dendritic spines in (C) CTR and (D) CAP1-KO neurons. GFP is shown in grayscale (single channel) or green (merge), phalloidin in ‘red hot’ (single channel) or cyan (merge) and Shank3 in grayscale (single channel) or magenta (merge). Scale bars (µm): 2 (A, B), 1 (C, D). Supplementary file7 (JPG 9147 KB) [file 18_2022_4593_MOESM7_ESM.jpg]

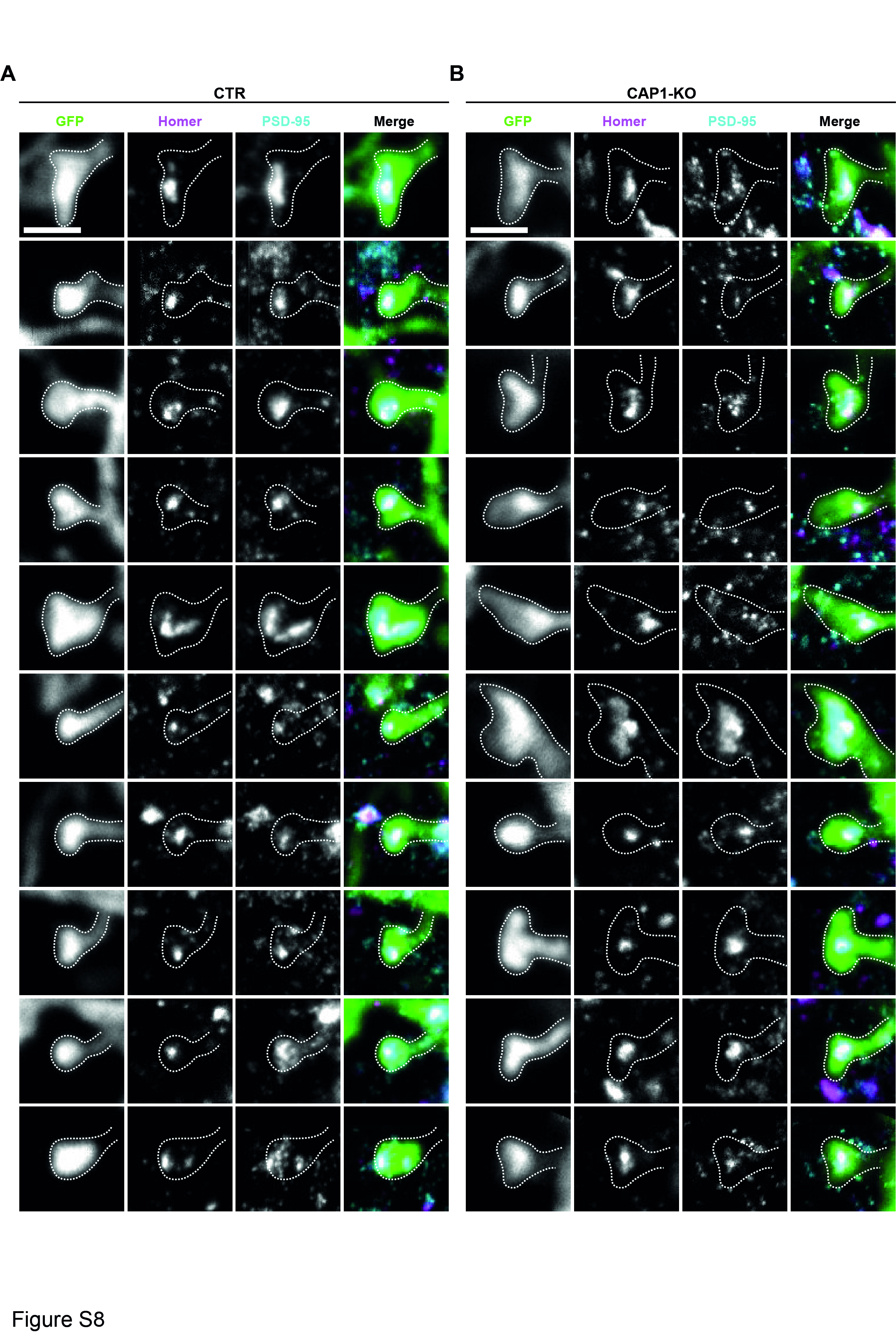

Supplement: Supplementary file 8 — Micrographs of dendritic spines from GFP-transfected (green) (A) CTR and (B) CAP1-KO neurons stained with antibodies against Homer (magenta) and PSD-95 (cyan). Scale bars (µm): 1 (A, B).Supplementary file8 (JPG 8761 KB) [file 18_2022_4593_MOESM8_ESM.jpg]

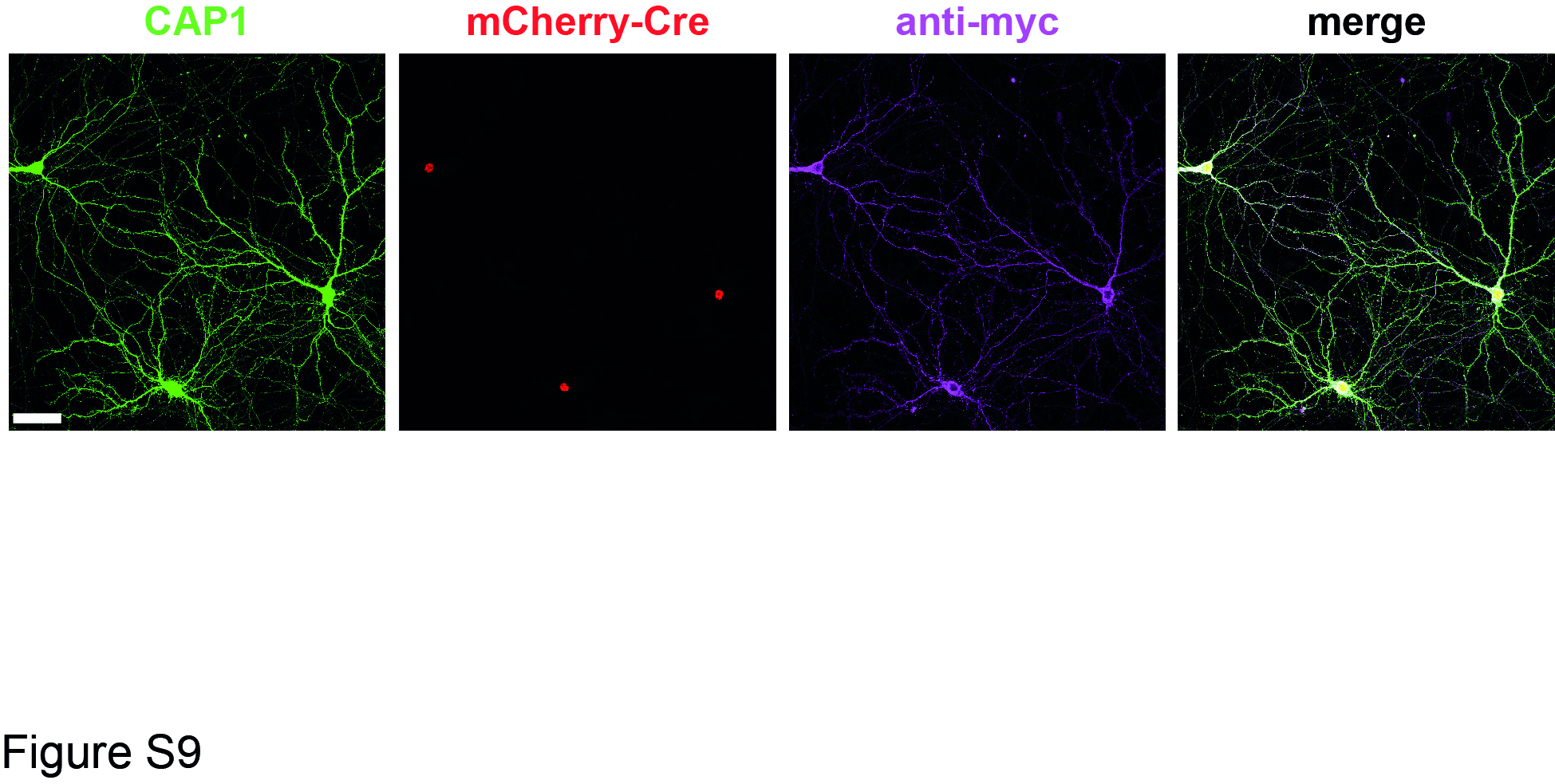

Supplement: Supplementary file 9 — Micrographs of DIV16 CAP1flx/flx neurons triple transfected at DIV6 with GFP (green), mCherry-Cre (red) and myc-WT-CAP1 (visualized by myc antibody staining (magenta)) demonstrating that neurons expressed all three transfected constructs. Scale bars (µm): 50. Supplementary file9 (JPG 2384 KB) [file 18_2022_4593_MOESM9_ESM.jpg]

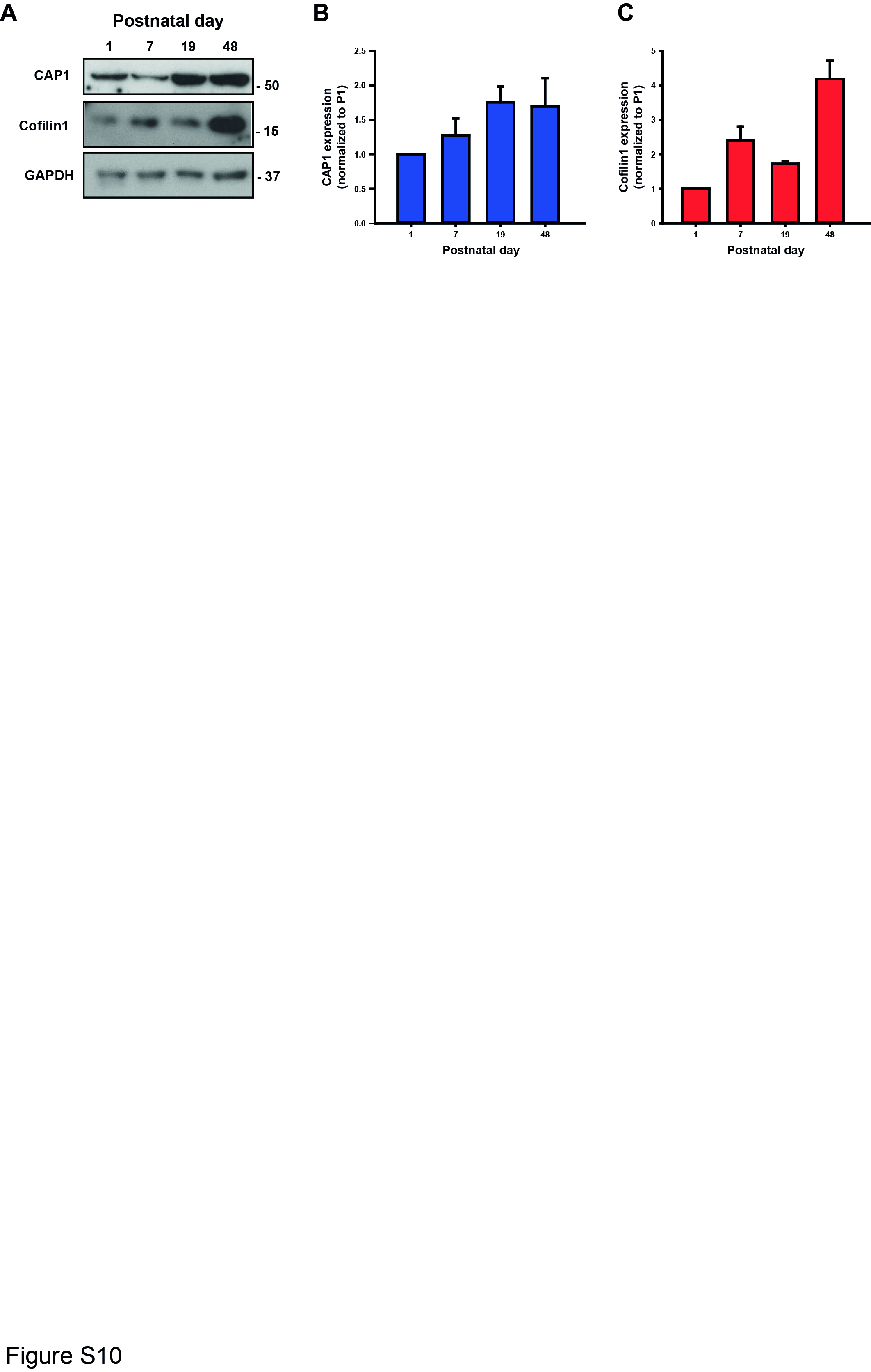

Supplement: Supplementary file 10 — (A) Immunoblots showing expression of CAP1 and cofilin1 at various postnatal stages in cerebral cortex. GAPDH was used as loading control and for normalization to protein load in statistical analyses. Graph showing expression levels of (B) CAP1 and (C) cofilin1 normalized to postnatal day 1 (P1). Supplementary file10 (JPG 816 KB) [file 18_2022_4593_MOESM10_ESM.jpg]

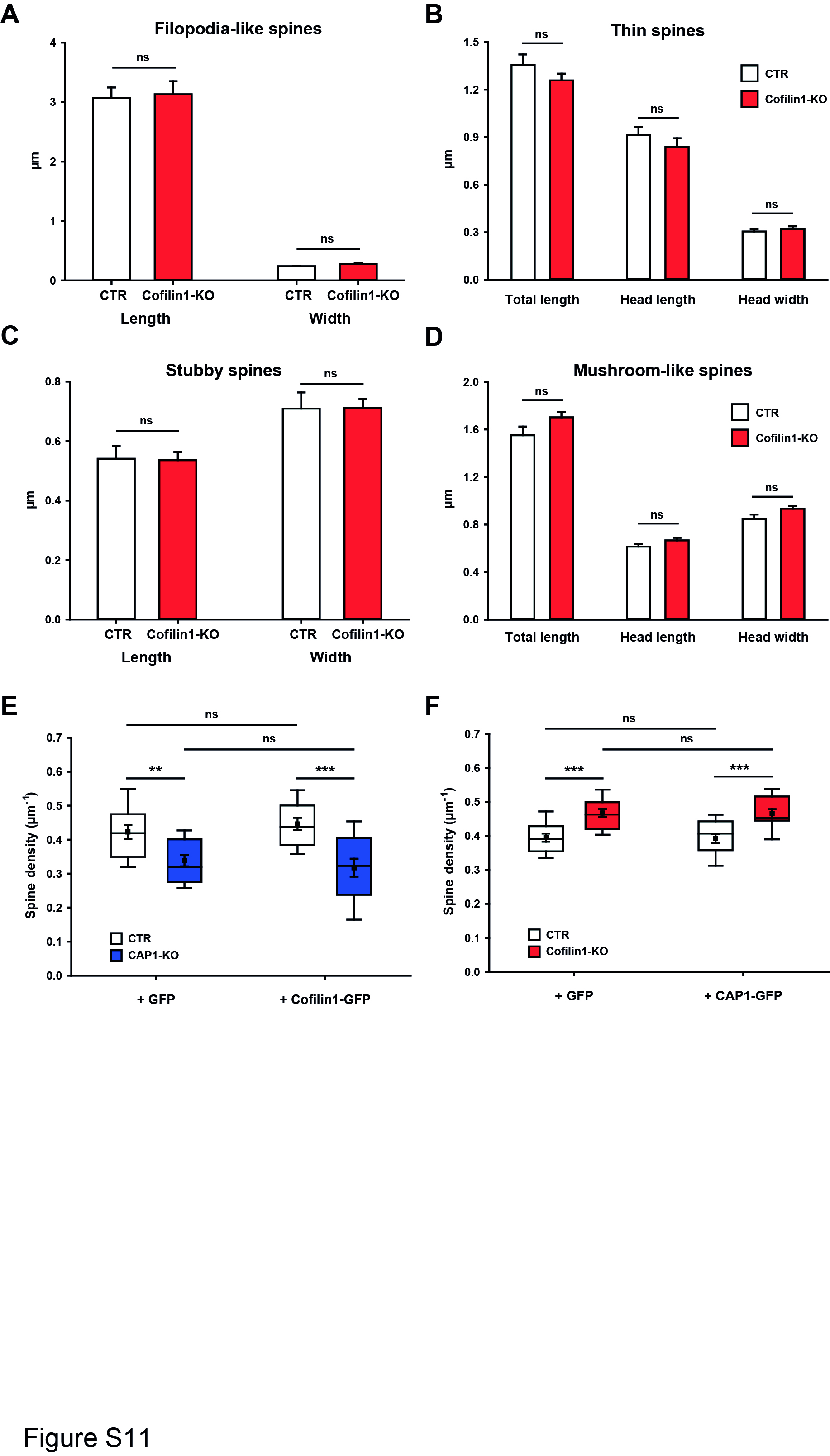

Supplement: Supplementary file 11 — Graphs showing (A) length and width of filopodia-like spines, (B) total length, head length and head width of thin spines, (C) length and width of stubby spines as well as (D) total length, head length and head width of mushroom-like spines in CTR and cofilin1-KO neurons. (E) Box plots (incl. MV±SEM) showing spine density in CTR and CAP1-KO neurons either expressing GFP or cofilin1-GFP. (F) Box plots (incl. MV±SEM) showing spine density in CTR and cofilin1-KO neurons either expressing GFP or CAP1-GFP. ns: P≥0.05, **: P<0.01. ***: P<0.001. Supplementary file11 (JPG 1375 KB) [file 18_2022_4593_MOESM11_ESM.jpg]

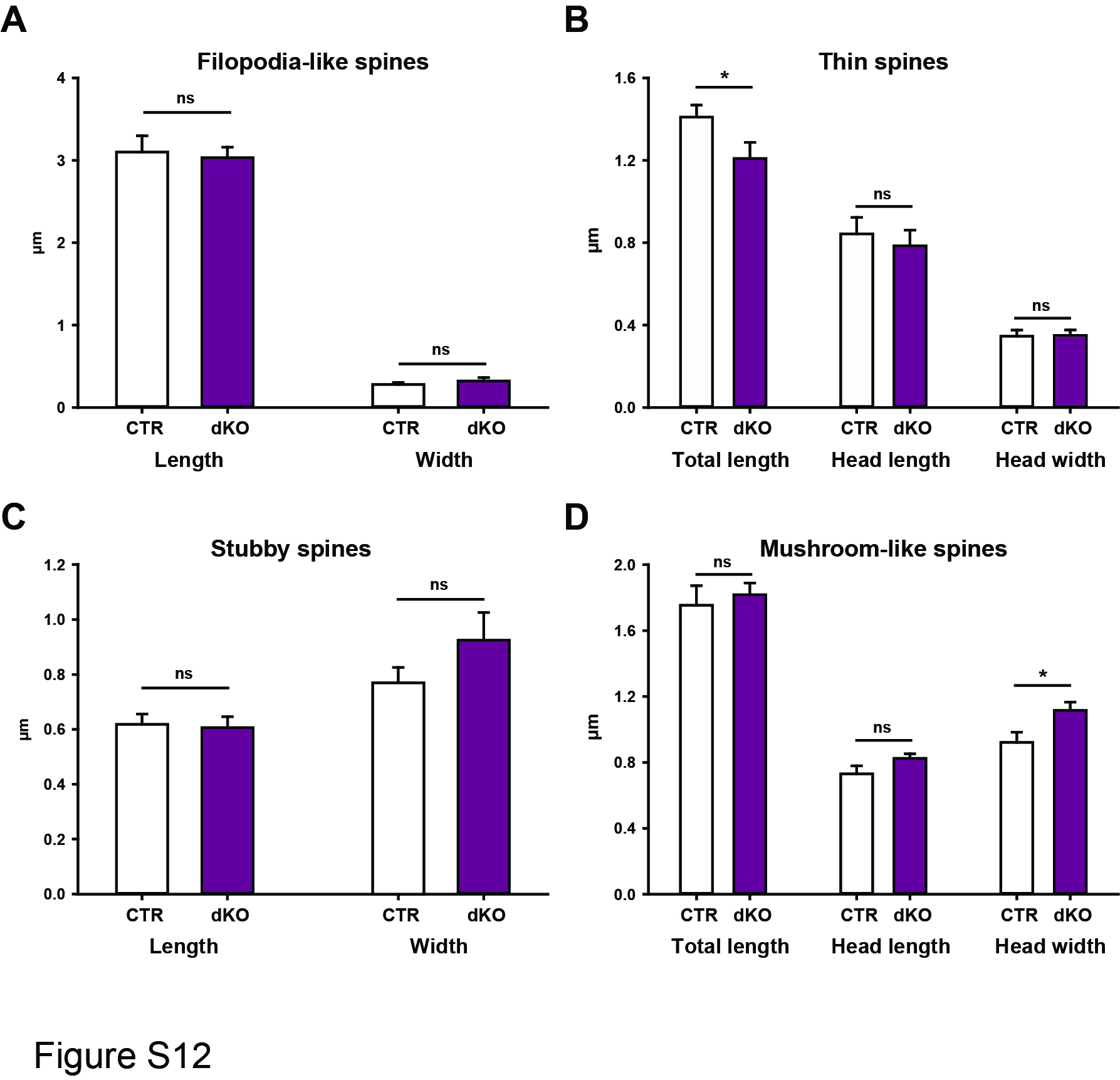

Supplement: Supplementary file 12 — Graphs showing (A) length and width of filopodia-like spines, (B) total length, head length and head width of thin spines, (C) length and width of stubby spines as well as (D) total length, head length and head width of mushroom-like spines in CTR and dKO neurons. ns: P≥0.05, *: P<0.05. Supplementary file12 (JPG 1510 KB) [file 18_2022_4593_MOESM12_ESM.jpg]
