# Supplementary material for: Functional interdependence of the actin regulators CAP1 and cofilin1 in control of dendritic spine morphology
Source: Cell Mol Life Sci. 2022 Oct 20;79(11):558. doi: 10.1007/s00018-022-04593-8 (PMC9585016; doi:10.1007/s00018-022-04593-8)
Supplement: Supplementary file 15 — Table showing parameters of dendritic morphology in CTR and CAP1-KO neurons as shown in Fig. S5. Significant changes between CTR and CAP1-KO are highlighted by colored font. Supplementary file15 (PDF 73 KB) [file 18_2022_4593_MOESM15_ESM.pdf]

Table S1

	CTR	CAP1-KO	P-Value (Change)
**Primary neurites**	5.79±0.49	6.04±0.53	0.731
**Branching points**	50.50±7.02	39.58±5.07	0.214
**Branching points/total length**	0.021±0.002	0.024±0.003	0.293
**Maximal intersections**	13.71±1.13	13.05±1.79	0.770
**Radius of maximal intersections**	**84.67±3.53**	**69.55±2.77**	**P<0.05 (-18%)**
**Ramification index**	2.52±0.24	2.49±0.20	0.921

N ≥ five CTR and CAP1-KO neurons from each experiment, three independent experiments
