# Supplementary material for: Functional interdependence of the actin regulators CAP1 and cofilin1 in control of dendritic spine morphology
Source: Cell Mol Life Sci. 2022 Oct 20;79(11):558. doi: 10.1007/s00018-022-04593-8 (PMC9585016; doi:10.1007/s00018-022-04593-8)
Supplement: Supplementary file 16 — Table showing spine density and volume in CAP1-KO and cofilin1-KO neurons and their corresponding CTR as shown in Figs. 2B-C and 6B-C. Moreover, the table includes spine density and volume in CTR and CAP1-KO neurons upon expression of myc-tagged CAP1 variants (named in left column) as shown in Fig. 4C-F. Significant changes are highlighted by colored font. Supplementary file16 (PDF 80 KB) [file 18_2022_4593_MOESM16_ESM.pdf]

Table S2

	Density		Volume	
	CTR	CAP1-KO	CTR	CAP1-KO
-	0.47±0.03	0.39±0.04<br>**-17% (P<0.01) to CTR**	0.22±0.01	0.28±0.03<br>**+27% (P<0.001) to CTR**
**+ WT-CAP1**	0.46±0.03<br>ns (P=0.971)<br>to CTR	0.47±0.05<br>**+21% (P<0.05) to CAP1-KO**	0.20±0.01,<br>ns (P=0.255)<br>to CTR	0.22±0.02<br>**-21% (P<0.01) to CAP1-KO**
**+ CAP1-HFD**	0.46±0.05<br>ns (P=0.939)<br>to CTR	0.38±0.03<br>ns (P=0.999)<br>to CAP1-KO	0.21±0.02<br>ns (P=0.770)<br>to CTR	0.27±0.02<br>ns (P=0.966)<br>to CAP1-KO
**+ CAP1-CARP**	0.47±0.03<br>ns (P=0.999)<br>to CTR	0.42±0.03<br>ns (P=0.797)<br>to CAP1-KO	0.24±0.01<br>ns (P=0.457)<br>to CTR	0.27±0.02<br>ns (P=0.883)<br>to CAP1-KO
**+ CAP1-P1**	0.48±0.03<br>ns (P=0.999)<br>to CTR	0.48±0.04<br>**+23% (P<0.05) to CAP1-KO**	0.22±0.02<br>ns (P=0.985)<br>to CTR	0.22±0.02<br>**-21% (P<0.01) to KO**
	CTR		Cofilin1-KO	
-	0.38±0.01	0.44±0.01<br>**+16% (P<0.001) to CTR**	0.23±0.01	0.32±0.01<br>**+39% (P<0.001) to CTR**

N ≥ 250 spines per neuron, five neurons per groups and experiment, three independent experiments
