# Supplementary material for: Functional interdependence of the actin regulators CAP1 and cofilin1 in control of dendritic spine morphology
Source: Cell Mol Life Sci. 2022 Oct 20;79(11):558. doi: 10.1007/s00018-022-04593-8 (PMC9585016; doi:10.1007/s00018-022-04593-8)
Supplement: Supplementary file 17 — Table summarizing morphometric analyses of spines (all spines, spine subtypes) in CAP1-KO, cofilin1-KO and dKO neurons and their corresponding CTR as shown in Figs. 2D-F, 3A, 6D-F, 7D-F, S6B-D, S11A-D and S12A-D. Significant changes are highlighted by colored font.Supplementary file17 (PDF 71 KB) [file 18_2022_4593_MOESM17_ESM.pdf]

Table S3

		CTR	CAP1-KO	P-Value (Change)	CTR	Cofilin1-KO	P-Value (Change)	CTR	dKO	P-Value (Change)
All	Length	1.51 ±0.10	1.42 ±0.09	0.488	1.42 ±0.08	1.46 ±0.05	0.643	1.62 ±0.08	1.56 ±0.07	0.553
	Head length	0.93 ±0.08	0.85 ±0.06	0.459	0.89 ±0.04	0.80 ±0.03	0.130	1.03 ±0.08	0.91 ±0.05	0.209
	Head width	**0.51 ±0.01**	**0.67 ±0.03**	**<0.001 (+31%)**	**0.59 ±0.02**	**0.71 ±0.02**	**<0.001 (+20%)**	**0.60 ±0.03**	**0.87 ±0.05**	**<0.001 (+45%)**
Filo-podia	Length	2.91 ±0.15	2.90 ±0.48	0.976	3.07 ±0.17	3.13 ±0.22	0.819	3.10 ±0.19	3.03 ±0.12	0.787
	Width	0.27 ±0.01	0.27 ±0.01	0.952	0.19 ±0.01	0.25 ±0.02	0.086	0.28 ±0.02	0.32 ±0.04	0.371
Thin	Length	1.28 ±0.05	1.13 ±0.07	0.100	1.36 ±0.06	1.26 ±0.04	0.190	**1.41 ±0.06**	**1.21 ±0.08**	**<0.05 (-14%)**
	Head length	0.80 ±0.05	0.79 ±0.05	0.933	0.91 ±0.05	0.84 ±0.05	0.307	0.84 ±0.0	0.79 ±0.07	0.607
	Head width	0.36 ±0.01	0.36 ±0.02	0.770	0.31 ±0.01	0.32 ±0.02	0.610	0.35 ±0.03	0.35 ±0.03	0.919
Stubby	Length	0.53 ±0.01	0.57 ±0.03	0.316	0.54 ±0.04	0.54 ±0.03	0.944	0.64 ±0.05	0.63 ±0.05	0.943
	Width	0.51 ±0.05	0.56 ±0.05	0.464	0.71 ±0.05	0.71 ±0.03	0.972	0.77 ±0.07	0.93 ±0.10	0.242
Mushroom	Length	1.59 ±0.09	1.69 ±0.11	0.510	1.55 ±0.07	1.70 ±0.04	0.085	1.75 ±0.12	1.82 ±0.07	0.649
	Head length	0.69 ±0.05	0.75 ±0.04	0.466	0.61 ±0.02	0.67 ±0.02	0.097	0.73 ±0.05	0.82 ±0.03	0.105
	Head width	**0.76 ±0.02**	**0.93 ±0.05**	**<0.01 (+22%)**	0.85 ±0.04	0.93 ±0.02	0.059	**0.92 ±0.06**	**1.12 ±0.05**	**<0.05 (+22%)**

N ≥ 15 spines per neuron, five neurons per group and experiment, three independent experiments
