# Supplementary material for: Functional interdependence of the actin regulators CAP1 and cofilin1 in control of dendritic spine morphology
Source: Cell Mol Life Sci. 2022 Oct 20;79(11):558. doi: 10.1007/s00018-022-04593-8 (PMC9585016; doi:10.1007/s00018-022-04593-8)
Supplement: Supplementary file 18 — Table summarizing spine type distribution in CAP1-KO, cofilin1-KO and dKO neurons and their corresponding CTR as shown in Figs. 2G, 6G, 7G. Significant changes are highlighted by colored font. Supplementary file18 (PDF 59 KB) [file 18_2022_4593_MOESM18_ESM.pdf]

Table S4

	CTR	CAP1-KO	Change	CTR	Cofilin1-KO	Change	CTR	dKO	Change
**Filopodia-like**	0.08 ±0.02	0.03 ±0.01	**-63%**	0.07 ±0.01	0.04 ±0.01	***-43%***	0.10 ±0.01	0.05 ±0.01	***-50%***
**Thin**	0.39 ±0.03	0.27 ±0.02	***-31%***	0.35 ±0.03	0.25 ±0.02	***-29%***	0.38 ±0.03	0.21 ±0.02	***-45%***
**Stubby**	0.13 ±0.01	0.22 ±0.02	***+69%***	0.11 ±0.02	0.16 ±0.01	***+45%***	0.12 ±0.02	0.18 ±0.02	***+50%***
**Mushroom-like**	0.29 ±0.02	0.37 ±0.02	***+28%***	0.40 ±0.05	0.50 ±0.02	***+25%***	0.32 ±0.03	0.51 ±0.02	***+59%***
**Branched**	0.09 ±0.01	0.05 ±0.01	***-44%***	0.04 ±0.01	0.02 ±0.01	***-50%***	0.06 ±0.02	0.04 ±0.01	***-33%***
**P-Value**		**<0.001**			**<0.01**			**<0.001**	

N ≥ 15 spines per neuron, five neurons per group and experiment, three independent experiments
