# Supplementary material for: Functional interdependence of the actin regulators CAP1 and cofilin1 in control of dendritic spine morphology
Source: Cell Mol Life Sci. 2022 Oct 20;79(11):558. doi: 10.1007/s00018-022-04593-8 (PMC9585016; doi:10.1007/s00018-022-04593-8)
Supplement: Supplementary file 19 — Table summarizing spine density and volume in CAP1-KO and cofilin1-KO neurons and their corresponding CTR before/after expression of either GFP-cofilin1 or and GFP-CAP1 (indicated in left column) as shown in Figs. 6J-K, S11E-F. Significant changes are highlighted by colored font. Supplementary file19 (PDF 78 KB) [file 18_2022_4593_MOESM19_ESM.pdf]

Table S5

	Density		Volume	
	**CAP1-CTR**	**CAP1-KO**	**CAP1-CTR**	**CAP1-KO**
	0.42±0.02	0.34±0.02<br>**-19% (P<0.01) to CAP1-CTR**	0.21±0.01	0.27±0.01<br>**+29% (P<0.01) to CAP1-CTR**
**+ WT-Cofilin1**	0.45±0.02<br>ns (P=0.399)<br>to CAP1-CTR	0.32±0.03<br>ns (P=0.512)<br>to CAP1-KO<br>**-24% (P<0.001) to CAP1-CTR**	0.19±0.01<br>ns (P=0.311)<br>to CAP1-CTR	0.28±0.01<br>ns (P=0.713)<br>to CAP1-KO<br>**+33% (P<0.001) to CAP1-CTR**
	**Cofilin1-CTR**		**Cofilin1-KO**	
**-**	0.40±0.01	0.47±0.01<br>**+18% (P<0.001) to Cofilin1-CTR**	0.20±0.01	0.27±0.01<br>**+35% (P<0.001) to Cofilin1-CTR**
**+ WT-CAP1**	0.40±0.01<br>ns (P=0.880)<br>to Cofilin1-CTR	0.47±0.01<br>ns (P=0.945)<br>to Cofilin1-KO<br>**+18% (P<0.001) to Cofilin1-CTR**	0.21±0.01<br>ns (P=0.748)<br>to Cofilin1-CTR	0.26±0.01<br>ns (P=0.166)<br>to Cofilin1-KO<br>**+30% (P<0.001) to Cofilin1-CTR**

N ≥ 250 spines per neuron, five neurons per group and experiment, three independent experiments
