# Supplementary material for: Functional interdependence of the actin regulators CAP1 and cofilin1 in control of dendritic spine morphology
Source: Cell Mol Life Sci. 2022 Oct 20;79(11):558. doi: 10.1007/s00018-022-04593-8 (PMC9585016; doi:10.1007/s00018-022-04593-8)
Supplement: Supplementary file 20 — Table summarizing changes in spine density and volume in CTR and dKO neurons before/after expression of CAP1 and cofilin1 constructs named in blue and red column as shown in Fig. 7I-J. Significant changes are highlighted by colored font. Supplementary file20 (PDF 78 KB) [file 18_2022_4593_MOESM20_ESM.pdf]

Table S6

			CTR	dKO
Density	-	-	0.39±0.01	0.36±0.01<br>**-8% (P<0.05) to CTR**
Volume	-	-	0.21±0.01	0.30±0.01<br>**+43% (P<0.001) to CTR**<br>ns (P=0.136) to CAP1-KO<br>ns (P=0.143) to Cofilin1-KO
	+ WT-CAP1	-	0.22±0.01,<br>ns (P=0.442) to CTR	0.30±0.01<br>ns (P=0.999) to dKO
	-	+ WT-Cofilin1	0.23±0.01<br>ns (P=0.174) to CTR	0.30±0.01<br>ns (P=0.999) to dKO
	+ WT-CAP1	+ WT-Cofilin1	0.22±0.01<br>ns (P=0.977) to CTR	0.24±0.01<br>**-20% (P<0.001) to dKO**
	+ CAP1-HFD	+ WT-Cofilin1	0.21±0.01<br>ns (P=0.989) to CTR	0.28±0.01<br>ns (P=0.509) to dKO
	+ CAP1-CARP	+ WT-Cofilin1	0.20±0.01<br>ns (P=0.656) to CTR	0.26±0.01<br>**-13% (P<0.05) to dKO**
	+ CAP1-P1	+ WT-Cofilin1	0.20±0.01<br>ns (P=0.979) to CTR	0.22±0.01<br>**-27% (P<0.001) to dKO**

N ≥ 250 spines per neuron, five neurons per group and experiment, three independent experiments
