# Supplementary material for: Functional interdependence of the actin regulators CAP1 and cofilin1 in control of dendritic spine morphology
Source: Cell Mol Life Sci. 2022 Oct 20;79(11):558. doi: 10.1007/s00018-022-04593-8 (PMC9585016; doi:10.1007/s00018-022-04593-8)
Supplement: Supplementary file 21 — List of oligonucleotides used for site-directed mutagenesis on CAP1 constructs. Supplementary file21 (PDF 223 KB) [file 18_2022_4593_MOESM21_ESM.pdf]

Table S7: List of oligonucleotides

			Oligonucleotides for cloning	
		Origin	forward	reverse
1	pcDNA3.1-CAP1-eGFP	Genescript	NA	NA
2	pEGFP-C1-CAP1	pcDNA3.1-CAP1-eGFP	(XhoI +) CTAGCTCGAGCGATGGCTGACATGCAAAATCTTG	(EcoRI +) CTAGGAATTCTTATCCAGCGATTTCTGTCACTG
3	pmCherry-C1-CAP1	pcDNA3.1-CAP1-eGFP	(XhoI +) CTAGCTCGAGCGATGGCTGACATGCAAAATCTTG	(BamHI +) CTAGGATCCTTATCCAGCGATTTCTGTCACTG
4	pCMV-Myc-N-CAP1	pcDNA3.1-CAP1-eGFP	(SalI +) CTAGTCGACTATGGCTGACATGCAAAATCTTGTAG	(NotI +) CTAGCGGCCGCTTATCCAGCGATTTCTGTCACTG
5	pCMV-Myc-N-CAP1-HFD	pCMV-Myc-N-CAP1	CTGTCGCCGCGGCGTACAGAGATGTGGATAAGAAGCATG	GCCGCGGCGACAGCATTTGTGTAAAACATGGCCGC
6	pCMV-Myc-N-CAP1-P1	pCMV-Myc-N-CAP1	CCACCGGGCCCGGCTGCTGCCCCAATCTCTAC	GGCCCGGTGGGGCAGCAGCTGGGCCTGATC
7	pCMV-Myc-N-CAP1-CARP	pCMV-Myc-N-CAP1	CAATTGCCTCCATTACAGTAGCTAACTGTAAGAAGCTTGGCCTGG	CTACTGTAATGGAGGCAATTGCGCCCTTGATTTGCAATGTTG
8	pcDNA3.1-CAP1-eGFP-HFD	pcDNA3.1-CAP1-eGFP	CTGTCGCCGCGGCGTACAGAGATGTGGATAAGAAGCATG	GCCGCGGCGACAGCATTTGTGTAAAACATGGCCGC
9	pcDNA3.1-CAP1-eGFP-P1	pcDNA3.1-CAP1-eGFP	CCACCGGGCCCGGCTGCTGCCCCAATCTCTAC	GGCCCGGTGGGGCAGCAGCTGGGCCTGATC
10	pcDNA3.1-CAP1-eGFP-CARP	pcDNA3.1-CAP1-eGFP	CAATTGCCTCCATTACAGTAGCTAACTGTAAGAAGCTTGGCCTGG	CTACTGTAATGGAGGCAATTGCGCCCTTGATTTGCAATGTTG
