# Supplementary material for: Functional interdependence of the actin regulators CAP1 and cofilin1 in control of dendritic spine morphology
Source: Cell Mol Life Sci. 2022 Oct 20;79(11):558. doi: 10.1007/s00018-022-04593-8 (PMC9585016; doi:10.1007/s00018-022-04593-8)
Supplement: Supplementary file 22 — List of primary antibodies used for immunocytochemistry (ICC) and immunoblots (IB). Supplementary file22 (PDF 70 KB) [file 18_2022_4593_MOESM22_ESM.pdf]

Table S8: List of primary antibodies

Antibody	Species	Dilution ICC, IB	Supplier	Cat.#
**anti-GFP**	rabbit	1:1,000, N/A	Thermo Fisher Scientific	G10362
**anti-c-myc**	mouse	1:200, N/A	Thermo Fisher Scientific	13-2500
**anti-CAP1**	mouse	1:200, 1:1,000	Abnova	H00010487-M02
**anti-Dcx**	rabbit	1:500, N/A	Abcam	ab18723
**anti-bassoon**	rabbit	1:250, N/A	Synaptic systems	141013
**anti-shank3**	guinea pig	1:250, N/A	Synaptic systems	162 304
**anti-GAPDH**	mouse	N/A, 1:1,000	R&D System	MAB5718
**anti-β-tubulin**	mouse	N/A, 1:2,000	Millipore	MAB1637
**anti-synaptophys.**	rabbit	N/A, 1:2,000	Synaptic Systems	101002
**anti-PSD95**	mouse	N/A, 1:2,000	Thermo Fischer Scientific	MA1-046
**anti-GFP**	chicken	N/A, 1:5,000	Abcam	Ab13970
