# Supplementary material for: Functional interdependence of the actin regulators CAP1 and cofilin1 in control of dendritic spine morphology
Source: Cell Mol Life Sci. 2022 Oct 20;79(11):558. doi: 10.1007/s00018-022-04593-8 (PMC9585016; doi:10.1007/s00018-022-04593-8)
Supplement: Supplementary file 23 — List of secondary antibodies used for immunocytochemistry (ICC) and immunoblots (IB). Supplementary file23 (PDF 324 KB) [file 18_2022_4593_MOESM23_ESM.pdf]

Table S9: List of secondary antibodies

Antibody	Species	Dilution ICC, IB	Supplier	Cat.#
**anti-mouse-AlexaFluor488**	donkey	1:2,000, N/A	Thermo Fisher Scientific	A-21202
**anti-mouse-AlexaFluor647**	donkey	1:2,000, N/A	Thermo Fisher Scientific	A-31571
**anti-rabbit-AlexaFluor546**	goat	1:2,000, N/A	Thermo Fisher Scientific	A-11035
**anti-rabbit-Alexa Fluor488**	goat	1:2,000/1:250 (STED), N/A	Thermo Fisher Scientific	A-11034
**anti-guinea pig-AlexaFluor488**	goat	1:250, N/A	Thermo Fisher Scientific	A-11073
**anti-guinea pig-Abberior635P**	goat	1:250, N/A	Abberior	st635P
**anti-mouse-AbberiorStar580**	goat	1:250, N/A	Abberior	2-0002-005-1
**anti-rabbit-HRP**	goat	N/A, 1:20,000	Thermo Fisher Scientific	31460
**anti-mouse-HRP**	goat	N/A, 1:20,000	Thermo Fisher Scientific	31430
**anti-mouse-IRDye**	goat	N/A, 1:20,000	Li-Cor	926-32210
**anti-rabbit-IRDye**	goat	N/A, 1:20,000	Li-Cor	926-32211
**Phalloidin-Atto647N**	N/A	1:40, N/A	Sigma	65906-10NMOL
**Hoechst**	N/A	1:20,000, N/A	Thermo Fisher Scientific	H1399
